# Biogenesis of Rab14-positive endosome buds at Golgi–endosome contacts by the RhoBTB3–SHIP164–Vps26B complex

**DOI:** 10.1038/s41421-024-00651-6

**Published:** 2024-04-02

**Authors:** Jingru Wang, Juan Xiong, Shuhan Zhang, Dongchen Li, Qingzhu Chu, Weiping Chang, Lin Deng, Wei-Ke Ji

**Affiliations:** 1 Department of Biochemistry and Molecular Biology, School of Basic Medicine, Huazhong University of Science and Technology, Wuhan, China; 2 Department of Ultrasound Medicine, Tangdu Hospital, Fourth Military Medical University, Xi’an, Shaanxi China; 3 Department of Anesthesiology and Pain Medicine, Hubei Key Laboratory of Geriatric Anesthesia and Perioperative Brain Health, and Wuhan Clinical Research Center for Geriatric Anesthesia, Tongji Hospital, Tongji Medical College, Huazhong University of Science and Technology, Wuhan, Hubei China; 4 Shenzhen Bay Laboratory, Shenzhen, Guangdong China; 5 Cell Architecture Research Center, Huazhong University of Science and Technology, Wuhan, Hubei China

**Keywords:** Retromer, Golgi

## Abstract

Early endosomes (EEs) are crucial in cargo sorting within vesicular trafficking. While cargoes destined for degradation are retained in EEs and eventually transported to lysosomes, recycled cargoes for the plasma membrane (PM) or the Golgi undergo segregation into specialized membrane structures known as EE buds during cargo sorting. Despite this significance, the molecular basis of the membrane expansion during EE bud formation has been poorly understood. In this study, we identify a protein complex comprising SHIP164, an ATPase RhoBTB3, and a retromer subunit Vps26B, which promotes the formation of EE buds at Golgi–EE contacts. Our findings reveal that Vps26B acts as a novel Rab14 effector, and Rab14 activity regulates the association of SHIP164 with EEs. Depletion of SHIP164 leads to enlarged Rab14^+^ EEs without buds, a phenotype rescued by wild-type SHIP164 but not the lipid transfer-defective mutants. Suppression of RhoBTB3 or Vps26B mirrors the effects of SHIP164 depletion. Together, we propose a lipid transport-dependent pathway mediated by the RhoBTB3–SHIP164–Vps26B complex at Golgi–EE contacts, which is essential for EE budding.

## Introduction

While vesicular transport modulates the bulk transport of many lipids, there is mounting evidence that lipid exchange facilitated by lipid transporters at membrane contact sites (MCSs) is the primary transport pathway for specific lipid types in given cellular contexts^1–5^, including organelle biogenesis^6–8^, organelle trafficking and division^9^, and overcoming lipotoxicity^10,11^.

Intracellular lipid transporters move lipids across opposing membranes at MCSs using shuttling or bridging modes^4,5,12^. Shuttle transporters often extract one or two lipid molecules from the membrane of the donor organelle, solubilize them during transport through the cytosol, and deposit them within the membrane of the acceptor organelle. In contrast, bridge transporters use an extended channel, typically lined with hydrophobic residues binding to tens of lipids simultaneously^5,13–15^. Several lipid transporter families, including synaptotagmin-like mitochondrial-lipid-binding domain-containing lipid transfer proteins, are shuttle transporters for glycerophospholipids and/or ceramides across MCSs in yeast and metazoans^11,16–21^. In contrast, the chorein family of lipid transporters, such as the Vps13 proteins and the autophagy-related protein Atg2, which have an extended hydrophobic groove along the protein structure, bind to dozens of lipids concurrently and belong to bridge lipid transporters^14,15,22–24^. Most of these bridge lipid transporters act as cytosolic proteins that are recruited to MCSs through two adapters localized on two opposing membranes of organelles. For instance, the mammalian Vps13 proteins are recruited to the endoplasmic reticulum (ER) via recognition of two phenylalanines in an acidic tract (FFAT) motif or phospho-FFAT motifs by ER adapters, such as vesicle-associated membrane proteins (VAPs)^25^ or motile sperm domain-containing protein 2^26^. In addition, these proteins are recruited to the other organelles by binding to other adapters using their C-terminal regions (CT)^15,22,27^. Recent evidence has demonstrated that the bridge lipid transporters enable lipid transfer to set the stage for the rapid growth of certain organelles^7^. Vps13 promotes the formation of the prospore membrane encapsulating the daughter nuclei, giving rise to spores in yeast^28^. Atg2 mediates the transport of phospholipids from the ER to growing autophagosomes during starvation^23,29^. Vps13B is necessary for acrosome biogenesis during sperm development in mice^30^, and Vps13D promotes peroxisome growth^31^.

In a pioneering study, Syntaxin 6 Habc domain-interacting protein with 164 kDa (SHIP164) was identified in a large (> 700 kDa) complex. SHIP164 has a Chorein N domain in its N-terminal region (NT), which is homologous to Vps13 proteins, and interacts with Syntaxin 6^32^. A recent study demonstrated that SHIP164 transfers glycerophospholipids in vitro, and localizes to clusters of small vesicles in the early endocytic pathway. Its depletion affects the size of early endosomes (EE) and impairs the retrograde trafficking of certain cargoes, indicating that SHIP164 is a bridge lipid transporter involved in transport from endosomes to the Golgi^33^. However, it remains unclear whether and how SHIP164 contributes to organelle/membrane structure biogenesis. In this study, we identified a protein complex containing SHIP164, an ATPase RhoBTB3, and a retromer subunit Vps26B, promoting the formation of EE buds at Golgi–EE contacts. We further identified Vps26B as a Rab14 effector, and Rab14 regulated the association of SHIP164 with EEs. Lipid transfer by SHIP164 was necessary for EE bud formation. Suppression of RhoBTB3 or Vps26B phenocopied the effects of SHIP164 depletion. Overall, we propose a lipid transport-dependent pathway mediated by the RhoBTB3–SHIP164–Vps26B complex at Golgi–EE contacts, which is essential for EE budding.

## Results

### SHIP164 cannot be recruited to the ER via VAPs

As a newly identified lipid transfer protein, the association between SHIP164 and MCSs remains enigmatic. Most lipids are synthesized in the ER^34^, and SHIP164-positive endocytic vesicles are localized adjacent to the ER^33^. Therefore, we examined whether SHIP164 could target the ER. Given that Vps13-related proteins target the ER via FFAT motifs, we therefore investigated whether SHIP164 was associated with the ER by this mechanism. According to the predicted algorithms for FFAT motifs^35,36^, several putative FFAT (Supplementary Fig. S1a) and phospho-FFAT motifs (Supplementary Fig. S1b) were identified in SHIP164, but they were weak. While co-immunoprecipitation (co-IP) assays demonstrated weak interactions between SHIP164 and the ER adapter VAPs at endogenous levels (Supplementary Fig. S1c), GFP-SHIP164 could not be recruited to the ER when the ER adapters were overexpressed (Supplementary Fig. S1d). Furthermore, coexpression of VAPB and SHIP164 phosphomimetic mutants, in which Ser and Thr were mutated to Asp in the core of these predicted phospho-FFAT motifs, did not result in recruitment of GFP-SHIP164 to the ER (Supplementary Fig. S1e). These findings indicated that the SHIP164–VAPs interaction was not sufficient for mediating stable recruitment of SHIP164 to the ER. Of note, we could not rule out the possibility that SHIP164 could be recruited to the ER by other unknown mechanisms.

### SHIP164 interacts with RhoBTB3 at Golgi–EE contacts

Bridge lipid transporters function at MCSs, with each terminus recognizing the two opposing membranes using two different adapters^37^. Because SHIP164 was not stably linked to the ER, we hypothesized that SHIP164 may associate with other membranes via an unknown adapter. To identify the adapter, we conducted a screen of small GTPase libraries, in which GFP-SHIP164 was co-expressed with each known GTPase, and examined the colocalization between GFP-SHIP164 and each small GTPase in human embryonic kidney 293 (HEK293) cells using live-cell confocal microscopy (Supplementary Fig. S2a). We employed HEK293 cells for this purpose, because they were more suitable for transfection and expression of the large SHIP164 construct (~10 kb).

**Fig. 1:**
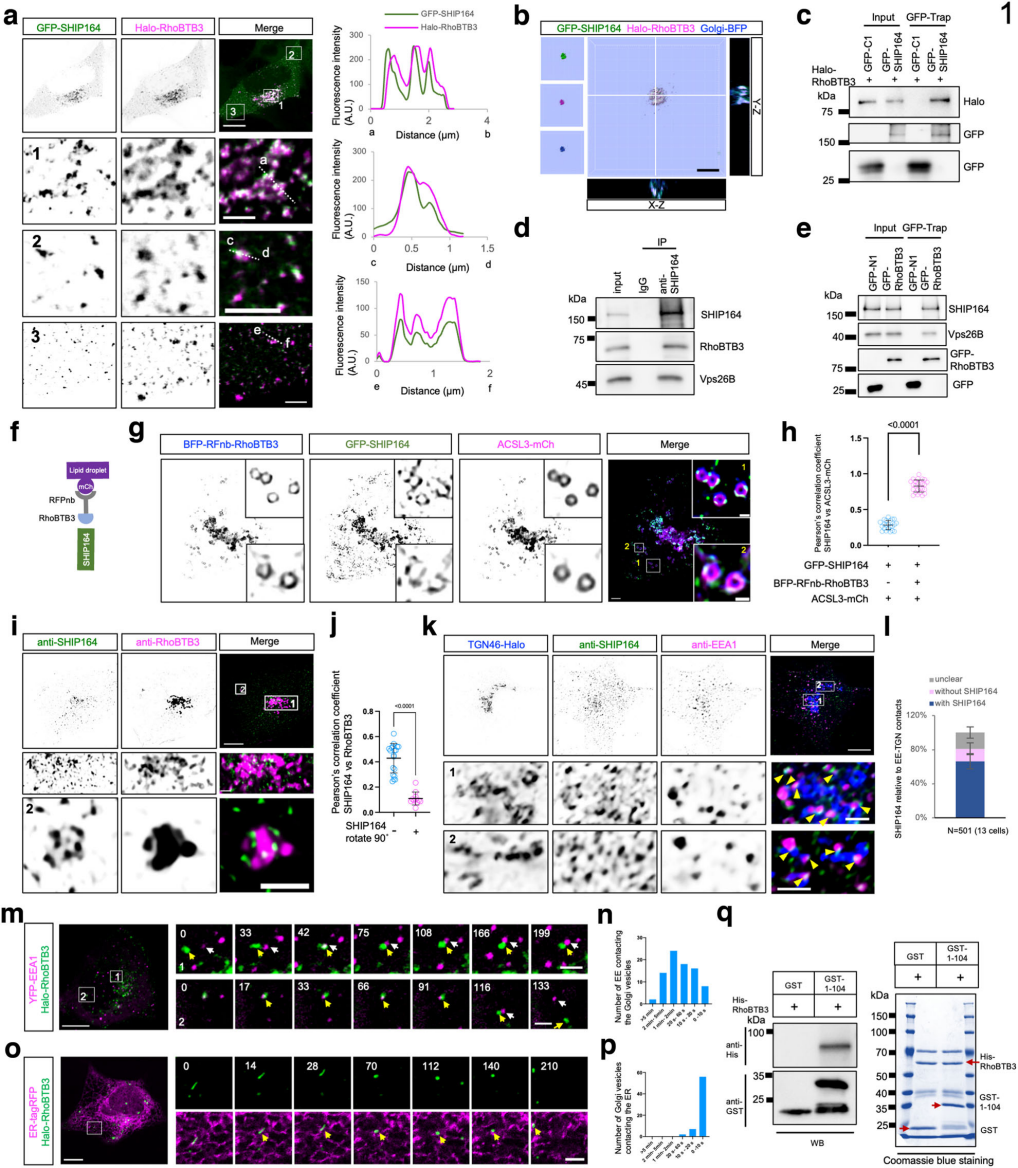
SHIP164 interacts with RhoBTB3. **a** Representative images of a live HEK293 cell expressing GFP-SHIP164 (green) and Halo-RhoBTB3 (magenta). Enlarged images of three boxed regions from the whole-cell image were shown on the bottom with line-scan analyses on the right. **b** Representative 3D rendering of a HEK293 cell expressing GFP-SHIP164 (green), Halo-RhoBTB3 (magenta), and Golgi-BFP (TM of B4GALT1) (blue) with *y*–*z* projection to the right and *x*–*z* projection to the bottom. **c** GFP-Trap assays demonstrate an interaction between GFP-SHIP164 and Halo-RhoBTB3 in HEK293 cells. **d** Co-IP assays show interactions between endogenous SHIP164 and endogenous RhoBTB3 or Vps26B in HEK293 cells. **e** Co-IP assays show interactions of endogenous SHIP164 and endogenous Vps26B with GFP-RhoBTB3 in HEK293 cells. **f** Schematic cartoon of RFPnb-mediated recruitment of BFP-RFPnb-RHOBTB3 to lipid droplet membranes (ACSL3-mCh) in the presence of GFP-SHIP164. **g** Representative images of a HEK293 cell expressing BFP-RFPnb-RhoBTB3 (blue), GFP-SHIP164 (green) and ACSL3-mCh (magenta) with insets. **h** Pearson’s correlation coefficient of GFP-SHIP164 vs ACSL3-mCh either in the presence (22 cells) or the absence (25 cells) of BFP-RFPnb-RHOBTB3. *n* > 3 independent experiments. Two-tailed unpaired Student’s *t*-test. Data are represented as mean ± SD. **i** Representative images of a fixed HEK293 cell stained with pre-cleared SHIP164 antibody and RhoBTB3 antibody (magenta) with two insets on the bottom. **j** Pearson’s correlation coefficient of endogenous SHIP164 puncta and RhoBTB3 (19 cells). Two-tailed unpaired Student’s *t*-test. Data are represented as mean ± SD. **k** Representative images of a fixed HEK293 cell expressing TGN46-Halo (blue) stained with pre-cleared SHIP164 antibody (green) and EEA1 antibody (magenta) with two insets on the bottom. **l** The distribution of endogenous SHIP164 puncta relative to TGN–EE junctions. 501 SHIP164 puncta and 743 potential TGN-EE junctions from 13 cells. Data are represented as mean ± SD. **m** Representative images of a HEK293 cell expressing YFP-EEA1 (magenta) and Halo-RhoBTB3 (green). Left: whole cell images; right: time-lapse images showing dynamic interactions between EEs and RhoBTB3-labled Golgi vesicles (Supplementary Videos S1, S2: time interval: 8 s). **n** Duration of the contacts between EEA1-labeled EEs and RhoBTB3 Golgi vesicles from 3 independent assays. **o** Representative images of a HEK293 cell expressing ER-tagRFP (magenta) and Halo-RhoBTB3 (green) (Supplementary Video S3, time interval: 14 s). **p** Duration of the contacts between the ER and RhoBTB3 Golgi vesicles from 3 independent assays. **q** GST-pull-down assays demonstrate that purified Chorein N domain of SHIP164, but not GST tag, was pelleted with purified His-RhoBTB3 in vitro. The Coomassie blue staining of proteins used in the assay was shown on the right. Red arrows denote the purified proteins in the gel. Scale bars: 10 μm in the whole cell images and 2 μm in the insets (**a**, **b**, **g**, **i**, **k**, **m**, **o**). Time shows in seconds.

We identified RhoBTB3, a peripheral Golgi protein functioning as an ATPase in retrograde trafficking^38,39^, as a novel SHIP164 interactor. This functionality was demonstrated by the recruitment of soluble GFP-SHIP164 to Halo-RhoBTB3 positive membranes (Fig. 1a; Supplementary Fig. S2b). This recruitment was verified using 3-dimensional (3D) reconstruction of *z*-stacks (Fig. 1b), as revealed by colocalization analyses based on *x*–*z* and *y*–*z* projections. An interaction between GFP-SHIP164 and Halo-RhoBTB3 was confirmed using GFP-Trap assays (Fig. 1c). Importantly, endogenous RhoBTB3 was co-IPed by endogenous SHIP164 (Fig. 1d). Because the RhoBTB3 antibody utilized in this study was unsuitable for co-IP, we performed GFP-Trap assays, finding that endogenous SHIP164 could also be co-IPed by GFP-RhoBTB3 (Fig. 1e), indicating a robust SHIP164–RhoBTB3 interaction. Both Halo-RhoBTB3 and endogenous RhoBTB3 were primarily localized on the *cis*/medial Golgi ribbon with a portion associated with *trans*-Golgi vesicles (Supplementary Fig. S2c, d).

To further confirm the SHIP164–RhoBTB3 interaction, we performed knock-sideway assays. Taking advantage of a specific interaction between RFP and an RFP nanobody (RFPnb), we ectopically targeted BFP-RFnb-RhoBTB3 to lipid droplet membranes marked by ACSL3-mCh (Fig. 1f). Remarkably, soluble GFP-SHIP164 was strongly recruited to lipid droplet membranes positive for RFnb-RhoBTB3 (Fig. 1g, h), suggesting a strong and direct interaction between SHIP164 and RhoBTB3.

We next assessed the spatial relationship between endogenous SHIP164 and RhoBTB3. Endogenous SHIP164 localized to clusters of endocytic vesicles^33^, but not the Golgi where RhoBTB3 was resident. We hypothesized that these two proteins interacted with each other only when a subset of EEs formed contacts with the Golgi. Indeed, immunofluorescence (IF) images demonstrated that SHIP164 was not completely colocalized with RhoBTB3, but a fraction of endogenous SHIP164 puncta was associated with RhoBTB3-positive membranes (Fig. 1i), which was specific and not by chance because the rotation of the SHIP164 image by 90° relative to the RhoBTB3 image significantly lowered the association (Fig. 1j). Importantly, we observed that more than half of Golgi–EE junctions were marked by endogenous SHIP164 (Fig. 1k, l), suggesting that the SHIP164–RhoBTB3 interaction preferentially occurred at Golgi–EE contacts.

Next, we performed live-cell time-lapse imaging to track the dynamics of RhoBTB3-positive Golgi vesicles and EEs. One of the most noticeable features was that both RhoBTB3 vesicles and EEs were motile, and these two organelles transiently but frequently contacted each other in a “kiss and run” manner. Noteworthy, they did not fuse during the imaging time window (Fig. 1m; Supplementary Videos S1, S2). Video analyses showed that the majority of EEs contacted RhoBTB3 vesicles for ~60–120 s (Fig. 1n). In addition, these Golgi vesicles also transiently contacted the ER, with most contacts lasting less than 10 s (Fig. 1o, p; Supplementary Video S3).

### The Chorein N domain of SHIP164 is responsible for the interaction with RhoBTB3

We next investigated the mechanism underlying the interaction between SHIP164 and RhoBTB3. By dissecting SHIP164, we found that an NT region containing residues 1–104 (Chorein N domain) was cytosolic (Supplementary Fig. S3a), but was strongly recruited to RhoBTB3-positive membranes when Halo-RhoBTB3 was co-expressed (Supplementary Fig. S3b). Consistently, when the Chorein N domain was removed, SHIP164 was not recruited by Halo-RhoBTB3 (Supplementary Fig. S3c, d). In addition, imaging results confirmed that both full-length SHIP164 and the Chorein N domain of SHIP164 were simultaneously recruited by RhoBTB3 in the same cells (Supplementary Fig. S3e). These results demonstrated that the Chorein N domain of SHIP164 was responsible for its recruitment by RhoBTB3.

To investigate whether SHIP164 interacted directly with RhoBTB3, we conducted in vitro pull-down assays. At this time, we were unable to produce purified full-length SHIP164 in sufficient quantity. Alternatively, we used purified Chorein N domain of SHIP164 and purified full-length RhoBTB3 in this assay. Indeed, GST-SHIP164-Chorein N, but not the GST tag, bound to His-RhoBTB3 (Fig. 1q). Overall, the result suggested that SHIP164 could bind to RhoBTB3 via the Chorein N domain.

### SHIP164 interacts with Vps26B

Because SHIP164 interacted with RhoBTB3 via its NT region, we wondered whether SHIP164 could bind to another protein on the other organelles via its CT region. To identify another adapter, we performed GST-pull-down assays followed by mass spectrometry (MS) utilizing purified GST-SHIP164-CT (residues 890–1464) as a bait in mouse brain lysates, in which SHIP164 was highly expressed (Supplementary Fig. S4a–c). After the removal of proteins co-pelleted by GST tags, we found several proteins functionally associated with the endosome-to-Golgi trafficking pathway (Supplementary Fig. S4d).

**Fig. 2:**
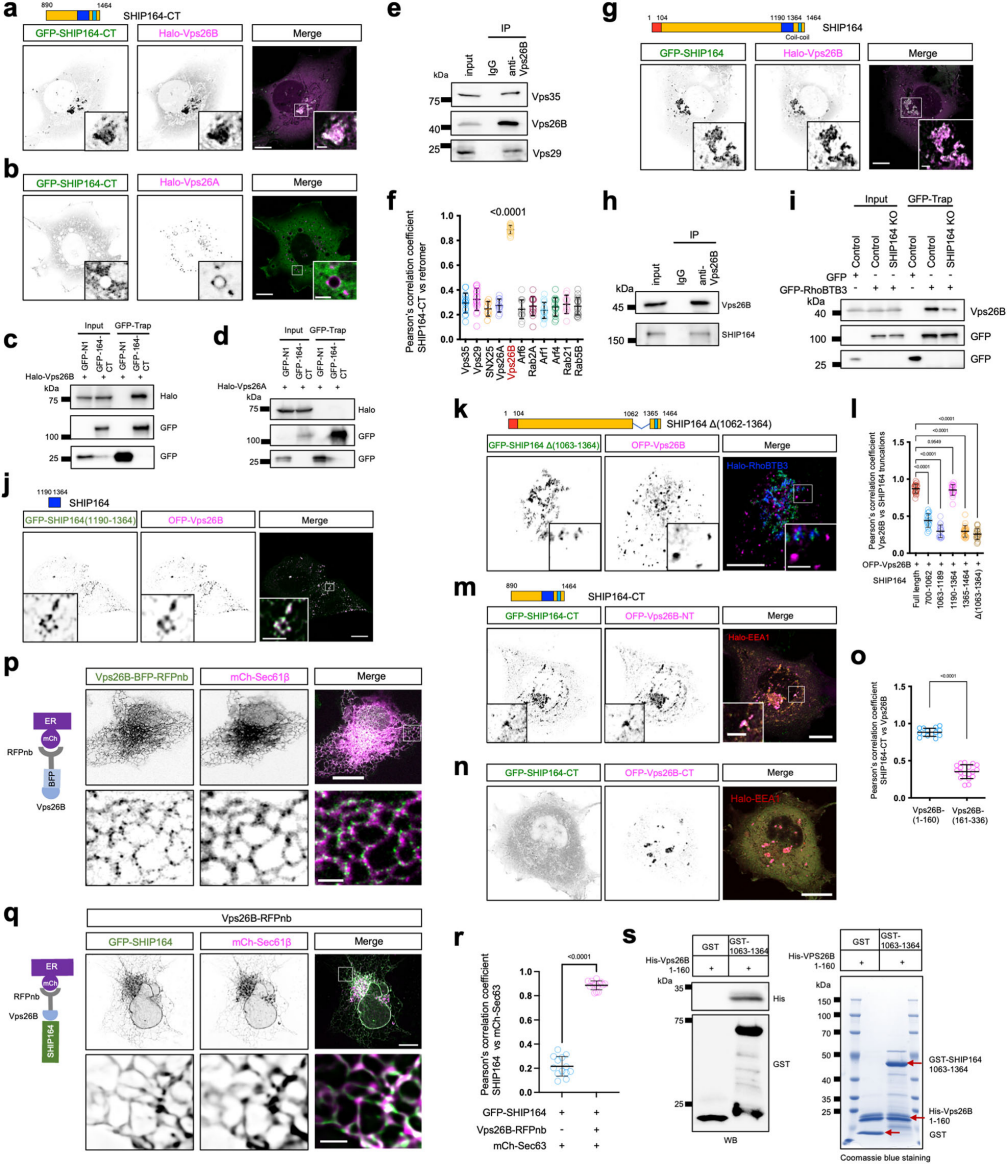
SHIP164 interacts with Vps26B. **a**, **b** Representative images of HEK293 cells expressing GFP-SHIP164-CT (green), along with either Halo-Vps26B (**a**, magenta) or Halo-Vps26A (**b**, magenta) with insets. **c**, **d** GFP-Trap assays demonstrate interactions between GFP-SHIP164-CT and Halo-Vps26B (**c**), but not Halo-Vps26A (**d**), in HEK293 cells. **e** Co-IP assays show interactions among Vps26B, Vps35, and Vps29 at the endogenous level in HEK293 cells. **f** Pearson’s correlation coefficient of GFP-SHIP164-CT vs either Halo-Arf6 (19 cells), Halo-Vps35 (16 cells), Halo-Rab2A (9 cells), Halo-Vps26A (12 cells), or Halo-Vps26B (28 cells), Halo-Arf1 (17 cells), Halo-Arf4 (15 cells), Halo-Rab21 (11 cells), Halo-Snx25 (9 cells), Halo-Rab5B (19 cells) or Halo-Vps29 (19 cells) in 3 independent experiments. Ordinary one-way ANOVA with Tukey’s multiple comparisons test. Data are represented as mean ± SD. **g** Representative images of a HEK293 cell expressing GFP-SHIP164 (green) and Halo-Vps26B (magenta) with insets. **h** Co-IP assays show interactions between endogenous Vps26B and endogenous SHIP164 in HEK293 cells. **i** GFP-Trap assays show interactions between endogenous Vps26B and GFP-RhoBTB3 in either control or *SHIP164* KO #9 HEK293 cells. **j**, **k** Representative images of a HEK293 cell expressing either GFP-SHIP164 (1190–1364, **j**), or SHIP164-Δ (1063–1364, green, **k**), and OFP-Vps26B (magenta) with insets. **l** Pearson’s correlation coefficient of Vps26B vs either full-length GFP-SHIP164 (22 cells), GFP-SHIP164 (700–1062), 18 cells), GFP-SHIP164 (1063–1189, 19 cells), GFP-SHIP164 (1190–1364, 19 cells), GFP-SHIP164(1365–1464, 20 cells), or GFP-SHIP164-Δ (1063–1364, 23 cells) in more than three independent experiments. Ordinary one-way ANOVA with Tukey’s multiple comparisons test. Data are represented as mean ± SD. **m**, **n** Representative images of live HEK293 cells expressing GFP-SHIP164-CT (green) with either OFP-Vps26B-NT (residues 1–160, magenta, **m**) or OFP-Vps26B-CT (residues 161–338, magenta, **n**) along with and Halo-EEA1 (red) with insets. **o** Pearson’s correlation coefficient of GFP-SHIP164-CT vs either OFP-Vps26B-NT (14 cells), or OFP-Vps26B-CT (18 cells) in more than 3 independent experiments. Ordinary one-way ANOVA with Tukey’s multiple comparisons test. Data are represented as mean ± SD. **p** Schematic cartoon of RFPnb-mediated recruitment of Vps26B-BFP-RFPnb to mCh-Sec61β-labeled ER membranes (left). Representative images of a HEK293 cell expressing Vps26B-BFP-RFPnb (green) and mCh-Sec61β with insets on the bottom (right). **q** Schematic cartoon of RFPnb-mediated recruitment of Vps26B-RFPnb to the ER (mCh-Sec61β, magenta) in presence of GFP-SHIP164 (green). Representative images of a HEK293 cell expressing GFP-SHIP164 (green), Vps26B-RFPnb, and mCh-Sec61β (magenta) with insets on the bottom (right). **r** Pearson’s correlation coefficient of GFP-SHIP164 vs mCh-Sec61β either in the presence (14 cells) or the absence (13 cells) of Vps26B-RFPnb. More than three independent experiments. Ordinary one-way ANOVA with Tukey’s multiple comparisons test. Data are represented as mean ± SD. **s** GST pull-down assays demonstrate that purified SHIP164-CT (residues 1063–1364), but not GST tag, was pelleted with purified His-Vps26B-NT (residues 1–160) in vitro. Coomassie blue staining of proteins used in the assay was shown on the right. Red arrows denote the purified proteins in the gel. Scale bars: 10 μm in the whole cell images and 2 μm in the insets (**a**, **b**, **g**, **j**, **k**, **m**, **n**, **p**, **q**).

We then examined the relationships between SHIP164-CT and these protein candidates using live-cell confocal microscopy. We identified Vps26B, a retromer subunit, as another SHIP164 interacting protein. The retromer is a protein coat that mediates the sorting and transport of endosomal proteins on endosome buds, followed by forming endosomal tubules recycling certain transmembrane proteins to the plasma membrane (PM) or the Golgi^40–43^. Notably, Vps26B, but not its paralog Vps26A^44,45^, recruited soluble GFP-SHIP164-CT (Fig. 2a, b), which was further confirmed by GFP-trap assays (Fig. 2c, d). While Vps26B interacted with Vps35 and Vps29 in the retromer complex (Fig. 2e), Vps29 or Vps35, as well as candidates shown in Supplementary Fig. S4d, did not recruit SHIP164-CT (Fig. 2f; Supplementary Fig. S4e). This indicated that the recruitment of SHIP164 was specific to Vps26B. We further confirmed that Vps26B could recruit full-length SHIP164 (Fig. 2g). Importantly, endogenous Vps26B could be co-IPed by endogenous SHIP164 (Fig. 1d). Meanwhile, endogenous SHIP164 could also be co-IPed by endogenous Vps26B (Fig. 2h), which indicated a strong interaction between SHIP164 and Vps26B under endogenous conditions. Interestingly, endogenous Vps26B could be co-IPed by GFP-RhoBTB3 (Fig. 1e), and *SHIP164* knockout (KO) significantly lowered the interaction (Fig. 2i). These findings suggested that the RhoBTB3–Vps26B interaction was, at least in part, dependent on SHIP164, and further supported the idea that SHIP164 links RhoBTB3 to Vps26B in the complex at the endogenous level.

Next, we sought to understand the mechanism underlying the SHIP164–Vps26B interaction through the dissection of Vps26B and SHIP164-CT. Live-cell microscopy showed that a region of SHIP164-CT (residues 1190–1364) was recruited by Vps26B (Fig. 2j). In contrast, neither the remaining portions of SHIP164-CT (700–1062, 1063–1189 or 1365–1464) (Supplementary Fig. S4f) nor SHIP164 with a deletion of the region containing residues 1062–1364 could be recruited by OFP-Vps26B (Fig. 2k, l). While Vps26B-NT and Vps26B-CT displayed similar localization (Supplementary Fig. S4g, h), Vps26B-NT (residues 1–160), but not the CT (residues 161–336), was responsible for the recruitment of SHIP164-CT to EEA1-positive endosomes (Fig. 2m, n). This suggested a difference in the NT region for binding partners between these two Vps26 proteins alongside the known variability in the CT region responsible for cargo selection between Vps26A and Vps26B^44^.

We next performed knock-sideway assays to confirm the interaction between SHIP164 and Vps26B. In these assays, we ectopically targeted Vps26B-RFPnb to ER membranes (mCh-Sec61β). We initially confirmed that the Vps26B-BFP-RFPnb was efficiently recruited to the ER through the binding of RFPnb to mCh-Sec61β (Fig. 2p). Crucially, soluble GFP-SHIP164 was strongly recruited to ER membranes that were positive for Vps26B-RFPnb (Fig. 2q, r), indicating that the SHIP164–Vps26B interaction was strong enough to mediate the recruitment.

Next, we performed in vitro pull-down assays to investigate whether SHIP164 interacted directly with Vps26B. In this assay, we used a purified SHIP164-CT fragment (residues 1063–1364) and purified Vps26B-NT fragment (residues 1–160). GST-SHIP164-CT, but not the GST tag alone, bound to His-Vps26B NT region (Fig. 2s). Collectively, our results demonstrated that SHIP164 bound to RhoBTB3 via the Chorein N domain in the NT region; meanwhile it bound to Vps26B through the CT region, further supporting that RhoBTB3, SHIP164 and Vps26B formed a protein complex at the contacts.

### SHIP164 depletion reduces Golgi–EE interactions

**Fig. 3:**
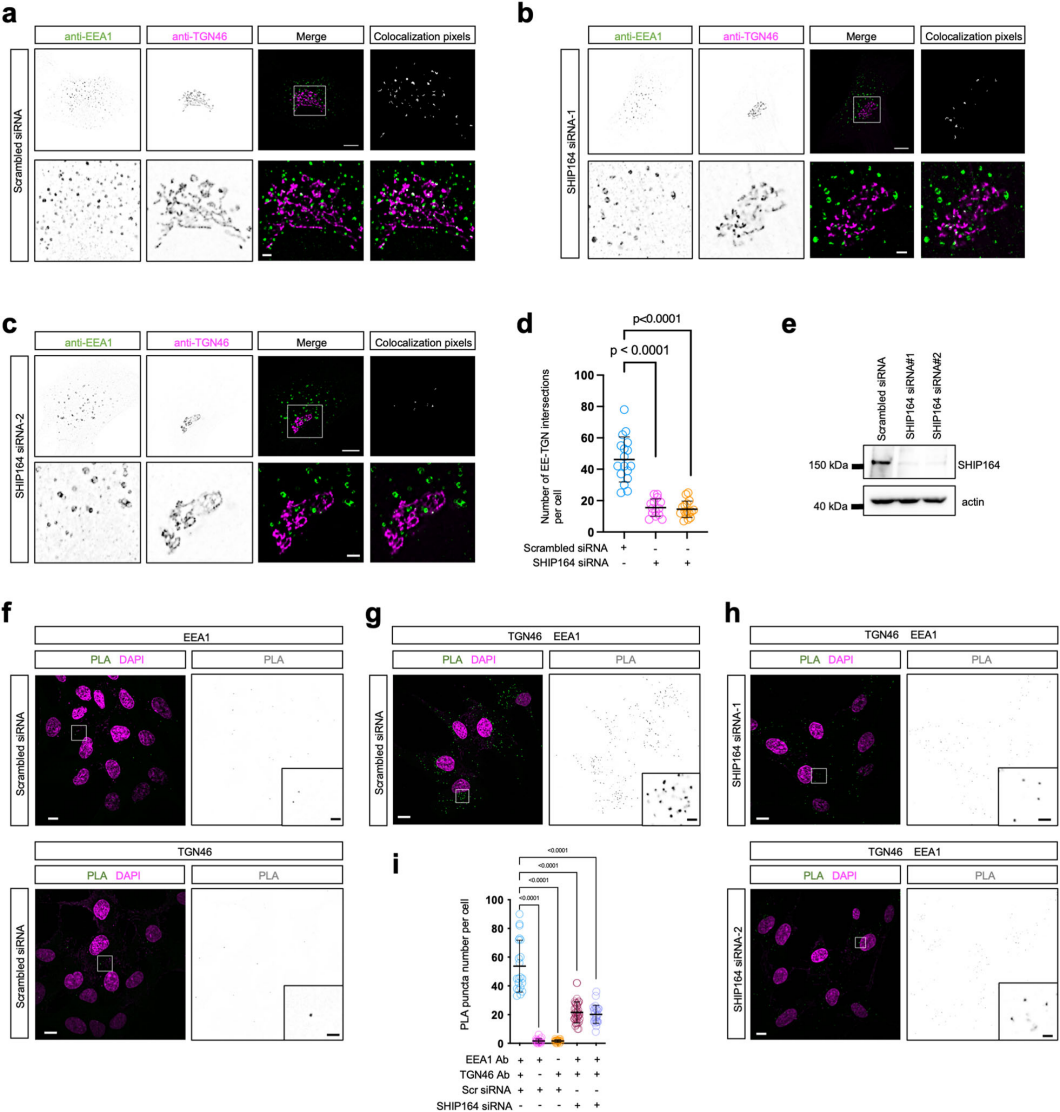
SHIP164 depletion reduces Golgi–EE interactions. **a**–**c** Representative images of fixed HEK293 cells labeling endogenous EEA1 (green) and TGN46 (magenta) with insets on the bottom were treated with either scrambled (**a**) or two *SHIP164* siRNAs (**b**, **c**). Overlapping pixels between anti-TGN46 and anti-EEA1 were shown on the right. **d** The number of Golgi–EE intersections per cell in scrambled (17 cells) or two independent *SHIP164* siRNA-treated cells (14 and 16 cells). Three independent assays were quantified for each condition. Two-tailed unpaired student’s *t*-test. Data are represented as mean ± SD. **e** Immunoblots showing the efficiency of siRNA-mediated depletion of SHIP164. **f**–**i** In situ proximity ligation assays to measure the effects of SHIP164 depletion on Golgi–EE associations in fixed HEK293 cells probed with primary antibodies (anti-EEA1 for EE; anti-TGN46 for the Golgi) followed by secondary antibodies coupled to specific oligonucleotides. EEA1 only (20 cells, **f** top panel) or TGN46 only (20 cells, **f**, bottom panel) were used as negative controls. Twenty-one scrambled cells and 40 *SHIP164* siRNA-1 or 26 *SHIP164* siRNA-2-treated cells were quantified from 3 independent experiments (**g**–**i**). Ordinary one-way ANOVA with Tukey’s multiple comparisons test. Data are represented as mean ± SD. Scale bars: 10 μm in the whole cell images and 2 μm in the insets (**a**–**c**, **f**–**h**).

The recruitment of SHIP164 to the Golgi and EEs via two distinct and distal regions suggested a tethering role of SHIP164 in mediating a novel type of MCSs between *trans*-Golgi and EEs. We explored the spatial relationship between the Golgi and EEs via IF staining in HEK293 cells. In control cells, a significant proportion of EEs (anti-EEA1) were closely associated with *trans*-Golgi (anti-TGN46), as revealed by a large number of EEs adjacent to the Golgi with overlapping pixels representing potential Golgi–EE contacts (Fig. 3a). SHIP164 depletion via two distinct small interfering RNAs (siRNAs) demonstrated that the association between *trans*-Golgi and EEs was significantly reduced, as illustrated by a reduction in the number of overlapping pixels between *trans*-Golgi and EEs (Fig. 3b, e).

**Fig. 4:**
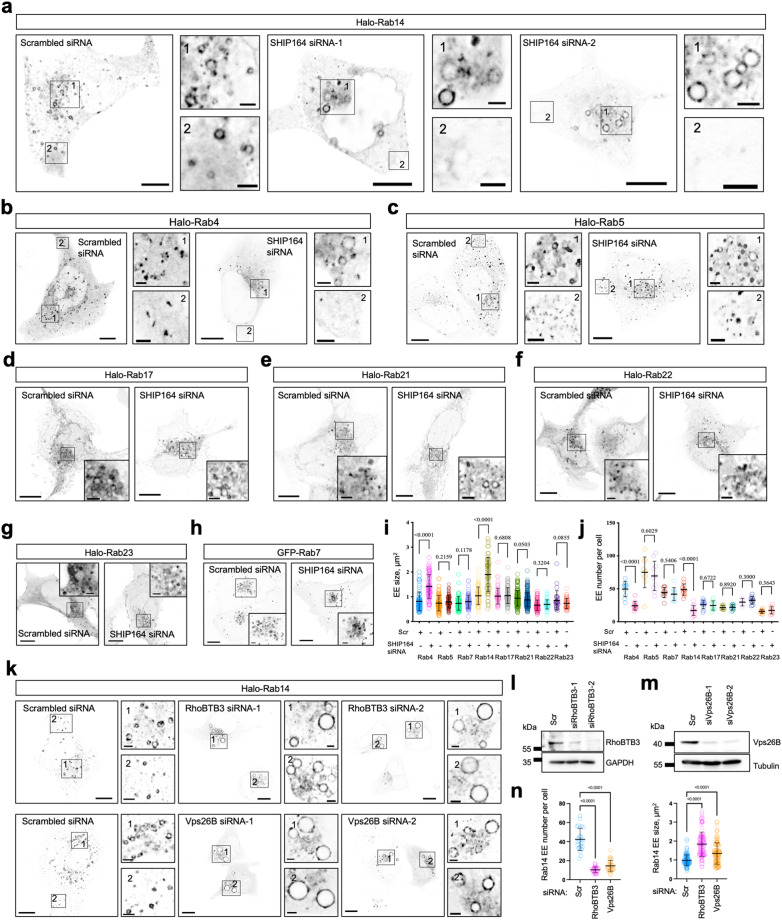
SHIP164 depletion strongly affects Rab14 EE, and RhoBTB3 or Vps26B depletion phenocopies the SHIP164 depletion. **a** Representative images of live HEK293 cells expressing Halo-Rab14 upon scrambled or two independent *SHIP164* siRNAs with two insets on the right. **b**–**h** Representative images of live HEK293 cells expressing multiple markers for endosomal subpopulations, including Halo-Rab4 (**b**), Halo-Rab5 (**c**), Halo-Rab17 (**d**), Halo-Rab21 (**e**), Halo-Rab22 (**f**), Halo-Rab23 (**g**), or GFP-Rab7 (**h**), upon scrambled or *SHIP164* siRNA with two insets, respectively. **i**, **j** The size (**i**) or number (**j**) of EEs per cell in scrambled or *SHIP164* siRNA-treated cells based on studies in **a**–**h**. More than 20 cells from three independent assays were quantified for each condition. Ordinary one-way ANOVA with Tukey’s multiple comparisons test. Data are represented as mean ± SD. **k** Representative images of live HEK293 cells expressing Halo-Rab14 upon two different siRNAs targeting RhoBTB3 (upper) or Vps26B (bottom) with two insets on the right. **l**, **m** Immunoblot analyses showing the efficiency of RhoBTB3 (**l**) or Vps26B (**m**) depletion via two siRNAs. **n** The number (left) or size (right) of EEs per cell in scrambled (26 cells), *RhoBTB3* (34 cells) or *Vps26B* (33 cells) siRNA-treated cells. Three independent assays were quantified for each condition. Ordinary one-way ANOVA with Tukey’s multiple comparisons test. Data are represented as mean ± SD. Scale bars: 10 μm in the whole cell images and 2 μm in the insets (**a**–**h**, **k**).

Notably, the Golgi–EE associations observed in IF staining may not indicate the existence of bona fide MCSs. Therefore, we performed in situ proximity ligation assays (PLA) to quantitatively examine the role of SHIP164 at the contacts. Contrary to PLA-negative controls, in which only one primary antibody was utilized (Fig. 3f), the Golgi and EEs indeed formed extensive contacts in scrambled siRNA-treated cells in the presence of anti-TGN46 and anti-EEA1 antibodies, as revealed by a large number of PLA puncta found in the cytosol (Fig. 3g). Importantly, SHIP164 depletion caused an over twofold reduction in the number of PLA puncta (Fig. 3h, i). This indicated an important role of SHIP164 in the formation or maintenance of Golgi–EE contacts. The PLA puncta was specific to Golgi–EE contacts, as the majority of these PLA puncta were present at intersections between the Golgi (marked by Halo-RhoBTB3) and EEs (marked by OFP-Vps26B) (Supplementary Fig. S4i, j). Notably, SHIP164 depletion impacted the number and size of EEs (Fig. 4i, j), which may also contribute to the dramatic reduction in Golgi–EE contacts. Therefore, we could not rule out the possibility that, in addition to the tethering function, SHIP164 may also regulate Golgi–EE MCSs through the modulation of EE homeostasis.

### Depletion of SHIP164 impairs homeostasis of Rab14-positive endosomes

Bridge lipid transporters, including Vps13 proteins and Atg2, play key roles in de novo biogenesis of organelles by providing membrane lipids to meet demands during membrane expansion and growth^23,28,29,31,46,47^. The bridge transporter SHIP164 is associated with endosomes, which led us to ask whether SHIP164 is required for endosome biogenesis. Because of the heterogeneity of endosomes (Supplementary Fig. S5a)^48^, we performed a miniscreen to examine the impacts of SHIP164 depletion on different endosomal subpopulations characterized by different Rab proteins in HEK293 cells. Depletion of SHIP164 influenced Rab14 (Fig. 4a) or Rab4-labeled endosomes (Fig. 4b), with Rab14-positive EEs being the most affected, compared to other Rabs, including Rab5 (Fig. 4c), Rab17 (Fig. 4d), Rab21 (Fig. 4e), Rab22 (Fig. 4f), Rab23 (Fig. 4g), and Rab7 (Fig. 4h). In addition, we found that the Golgi labeled by the GM130 antibody was not dramatically influenced by SHIP164 depletion (Supplementary Fig. S5b). Therefore, we focused on Rab14-positive endosomes in this study. The size of Rab14-positive endosomes was greatly increased (Fig. 4i) but the number was reduced (Fig. 4j) in SHIP164-depleted HEK293 cells. Rab14 is associated with a subpopulation of EE and relevant transport carriers and is involved in membrane traffic from endosomes to the Golgi^49^. Therefore, this finding suggested a role of SHIP164 in Rab14-positive endosomal homeostasis at EE-to-Golgi retrograde trafficking.

Intriguingly, depletion of RhoBTB3 or Vps26B via two different siRNAs resulted in fewer but larger Rab14-positive EE (Fig. 4k–n), phenocopying the SHIP164 depletion. These findings suggested that SHIP164, RhoBTB3, and Vps26B were functionally associated with Rab14-positive EE homeostasis.

Next, we investigated how SHIP164 depletion influenced the homeostasis of Rab14-positive EEs. Notably, the depletion of Rab5, EEA1, or expression of a dominant-negative Rab5 mutant Rab5 S34N significantly rescued the Rab14-positive EE phenotype in SHIP164-depleted cells (Supplementary Fig. S5c–e), with the number (Supplementary Fig. S5f) or size (Supplementary Fig. S5g) of Rab14-positive EE being restored. These findings suggested that the Rab14-positive EE phenotype may be due to the upregulation of Rab5 or EEA1 activities in SHIP164-depleted cells.

### Vps26B is a Rab14 effector at EE buds

Vps26B is a subunit of the retromer complex acting at the budding sites of endosomes, where endosomal tubules grow and fission^40,42,44,50–54^. Our findings demonstrated that Vps26B interacted with SHIP164, the depletion of which affected Rab14-positive endosomes, suggesting a possible link between Rab14 and Vps26B. To test this hypothesis, we first confirmed that endogenous Vps26B was associated with EEs (GFP-FYVEx2) to a higher extent than with late endosomes/lysosomes (anti-Lamp1) (Supplementary Figs. S5b, S6a). In contrast to the overexpressed Vps26B that decorated the entire EE membranes (Fig. 2a, g), endogenous Vps26B formed foci, and localized to specific microdomains of EEs, which were presumably the budding sites of endosomes (endosomal buds) (Supplementary Fig. S6a), consistent with the reported localization and function of the retromer^40,44,55^. The specificity of the Vps26B antibody in IF staining was confirmed by siRNA-mediated depletion (Supplementary Fig. S6b, c).

**Fig. 5:**
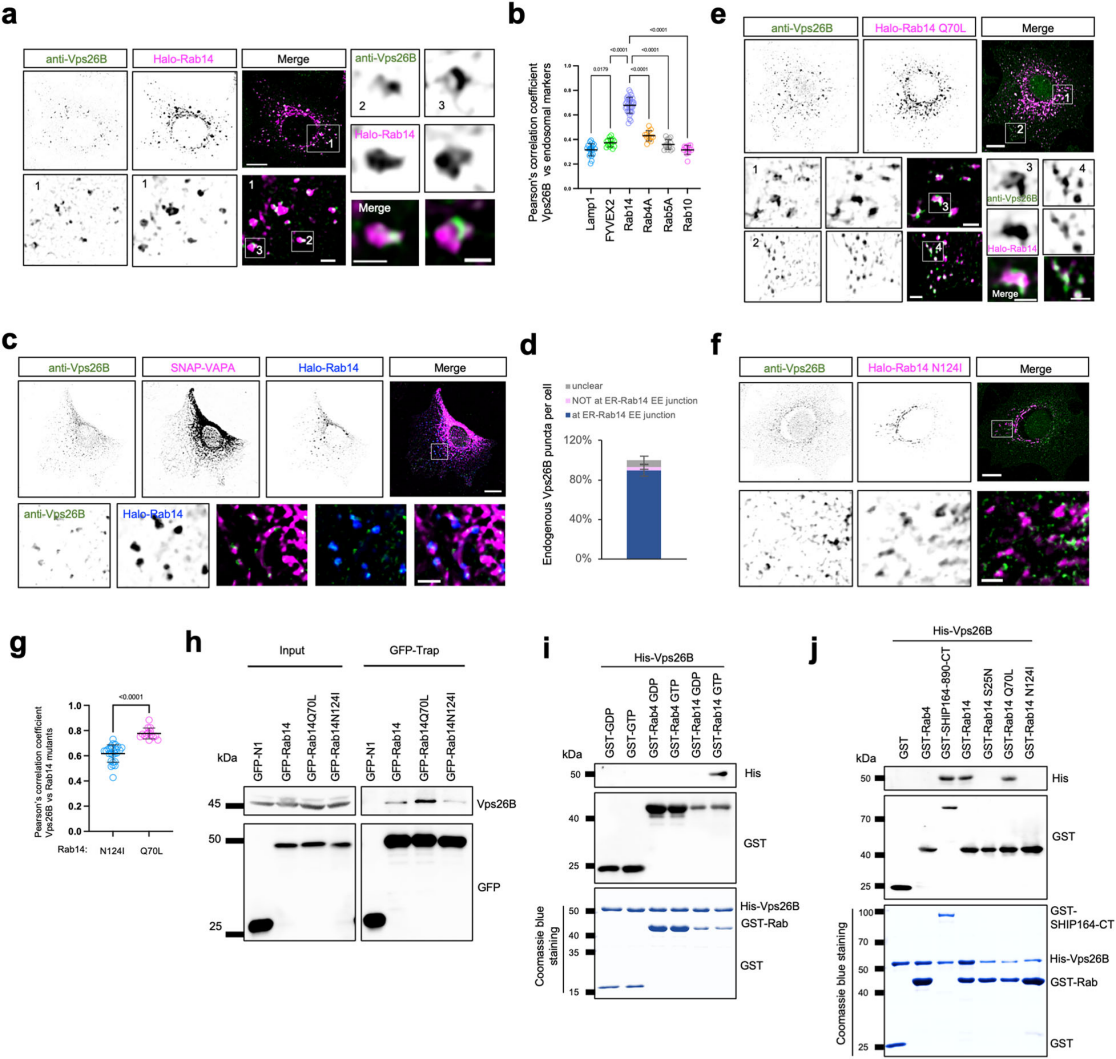
Vps26B is a Rab14 effector at EE buds. **a** Representative images of fixed HEK293 cells stained with Vps26B antibody (green) and expressing Halo-Rab14 (magenta) with one large inset on the bottom and two small insets on the right. **b** Pearson’s correlation coefficient of endogenous Vps26B vs endosomal markers: Lamp1 (20 cells), GFP-FYVEX2 (13 cells), Rab10 (12 cells), Rab4A (12 cells), Rab5A (14 cells), Rab14 (32 cells), or FYVEx2 (13 cells) in 3 independent experiments. Ordinary one-way ANOVA with Tukey’s multiple comparisons test. Data are represented as mean ± SD. **c** Representative images of fixed HEK293 cells stained with Vps26B antibody (green) and expressing Halo-Rab14 (blue) along with SNAP-VAPA (magenta). An inset was shown on the bottom with line-scan analyses on the right. **d** The distribution of endogenous Vps26B puncta relative to ER-Rab14 EE junctions. (1019 Vps26B puncta from 18 cells). Data are represented as mean ± SD. **e**, **f** Representative images of fixed HEK293 cells stained with Vps26B antibody (green) and expressing either Halo-Rab14 Q70L (magenta, **e**) or Halo-Rab14 N124I (magenta, **f**) with an inset on the bottom. **g** Pearson’s correlation coefficient of endogenous Vps26B vs either Rab14 N124I (26 cells) or Rab14 Q70L (14 cells), in more than three independent experiments. Two-tailed unpaired Student’s *t*-test. Data are represented as mean ± SD. **h** GFP-Trap assays demonstrate interactions between Halo-Rab14 mutants and endogenous Vps26B in HEK293 cells. **i** GST-pull-down assays show that GTP-loaded Rab14, but not Rab14-GDP, Rab4-GTP, or GST tag alone, binds to His-Vps26B. The Coomassie blue staining of proteins used in the assay is shown at the bottom. **j** GST-pull-down assays demonstrate that purified Rab14 and Rab14 Q70L, but not GST tag, or dominant-negative mutants S25N or N124I, were pelleted with purified His-Vps26B in vitro, with Rab4 as a negative control and SHIP164-890-CT as a positive control. The Coomassie blue staining of proteins used in the assay is shown at the bottom. Scale bars: 10 μm in the whole cell images and 2 μm in the insets (**a**, **c**, **e**, **f**).

Importantly, endogenous Vps26B was preferentially localized on Rab14 EE buds (Fig. 5a), but was not significantly associated with other endosomal subpopulations, such as Rab4A, Rab10, and Rab5A (Fig. 5b; Supplementary Fig. S6d). In addition, a substantial fraction (> 80%) of endogenous Vps26B foci localized to Rab14-positive endosome buds near the ER (Fig. 5c, d), consistent with previous studies^56,57^.

We next investigated whether Rab14 activity regulated the association of Vps26B with EEs. Remarkably, Rab14 Q70L, a constitutively active mutant, strongly enhanced the association between Vps26B and EEs, which resulted in the decoration of endogenous Vps26B across nearly the entire membranes of Rab14 Q70L-labeled endosomes (Fig. 5e). Conversely, an inactive mutant, Rab14 N124I, significantly lowered the association with endogenous Vps26B, compared to wild-type (WT) or the Rab14 Q70L mutant (Fig. 5f, g). Accordingly, co-IP showed that a higher level of endogenous Vps26B was co-IPed with GFP-Rab14 Q70L than either WT Rab14 or the N124I mutant (Fig. 5h). These findings indicated that Rab14 activity regulated the association of Vps26B with EE buds, which suggested that Vps26B may be an effector of Rab14.

To investigate whether Vps26B was indeed an effector of Rab14, we performed GST pull-down assays. We found that purified full-length His-Vps26B specifically interacted with the GTP-bound form of Rab14 but not GST tag alone, Rab14-GDP or GTP-loaded Rab4 (Fig. 5i). These findings were further confirmed by GST pull-down assays using purified Rab14 mutants. Purified His-Vps26B interacted directly with GST-tagged WT Rab14 or the constitutively active mutant Rab14 Q70L, but not GST alone or the two inactive mutants Rab14 N124I or S25N, and there was no interaction with GST-Rab4 (Fig. 5j). In addition, the pull-down assays confirmed that purified SHIP164-CT bound to full-length His-Vps26B (Fig. 5j). Collectively, our results indicated that Vps26B was a Rab14 effector at EE buds.

### Rab14 regulates the association of SHIP164 with EEs

**Fig. 6:**
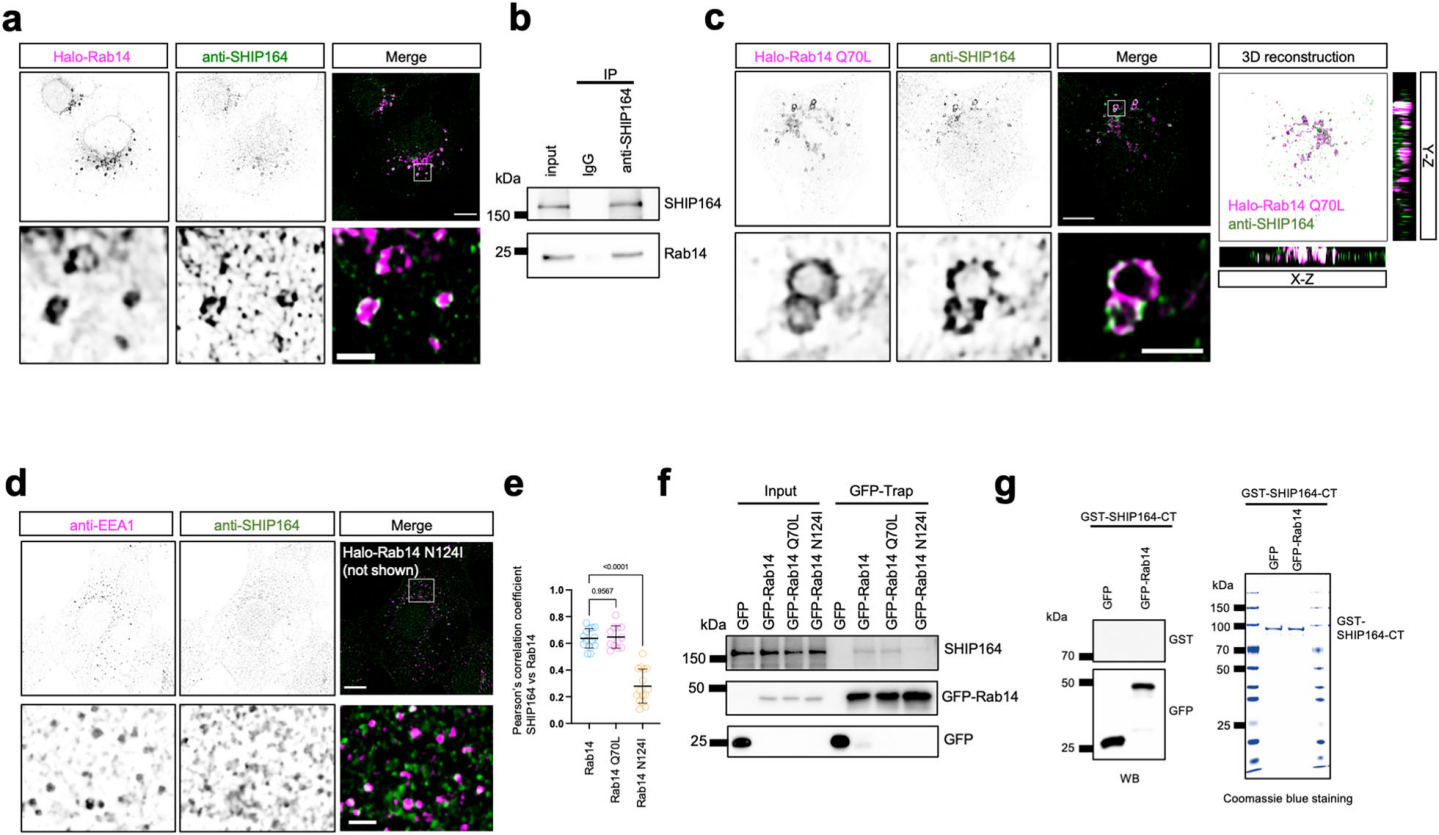
Rab14 regulates the association of SHIP164 with EEs. **a** Representative images of fixed HEK293 cells stained with SHIP164 antibody (green) and expressing Halo-Rab14 (magenta) with an inset on the bottom. **b** Co-IP assays show interactions between endogenous Rab14 and endogenous SHIP164 in HEK293 cells. **c** Representative images of fixed HEK293 cells expressing Halo-Rab14 Q70L (magenta) and stained with SHIP164 antibody (green) with an inset on the bottom (left). 3D rendering of a fixed HEK293 cell, with *y*–*z* projection to the right and *x*–*z* projection to the bottom. **d** Representative images of fixed HEK293 cells stained with SHIP164 antibody (green) and EEA1 antibody (magenta), along with the expression of Halo-Rab14 N124I (not shown in this image) with an inset on the bottom. **e** Pearson’s correlation coefficient of endogenous SHIP164 vs Rab14 (13 cells) and Rab14 mutants: Rab14 Q70L (11 cells), or Rab14 N124I (14 cells) in three independent experiments. Ordinary one-way ANOVA with Tukey’s multiple comparisons test. Data are represented as mean ± SD. **f** GFP-Trap assays demonstrate interactions between Halo-Rab14 mutants and endogenous SHIP164 in HEK293 cells. **g** Pull-down assays demonstrate that purified GST-SHIP164-CT did not bind to GFP-Rab14 in vitro. The Coomassie blue staining of GST-SHIP164-CT used in the assay was shown on the right. Scale bars: 10 μm in the whole cell images and 2 μm in the insets (**a**, **c**–**d**).

We next investigated whether Rab14 activity regulated the association of SHIP164 with EEs. Indeed, IF images showed that endogenous SHIP164 was strongly recruited to EEs when Halo-Rab14 was expressed (Fig. 6a). The interaction between SHIP164 and Rab14 was confirmed by endogenous co-IP assays (Fig. 6b). Consistently, the constitutively active mutant Halo-Rab14 Q70L also strongly recruited endogenous SHIP164 (Fig. 6c; left), which was confirmed by 3D images (Fig. 6c; right). In contrast, the recruitment of SHIP164 was substantially hindered upon expression of the negative Rab14 mutant N124I (Fig. 6d, e). Furthermore, co-IP confirmed that both WT and Rab14 Q70L interacted with endogenous SHIP164 to a greater extent than the Rab14 N124I mutant (Fig. 6f).

To examine whether SHIP164 could interact directly with Rab14, we conducted GFP-Trap assays to pellet GFP-Rab14 from HEK293 cells transiently expressing GFP-Rab14 using a high-salt (500 mM NaCl) lysis buffer, as previously described^21^. After rigorous washing to remove proteins that could co-pellet with GFP-Rab14 under high-salt conditions, purified GST-SHIP164-CT was incubated with GFP tag beads or GFP-Rab14 beads, respectively. The pull-down assays showed that purified SHIP164-CT did not bind to GFP-Rab14 (Fig. 6g), indicating that Rab14 activity indirectly regulated SHIP164 recruitment to EEs.

### SHIP164 is required for EE bud growth

**Fig. 7:**
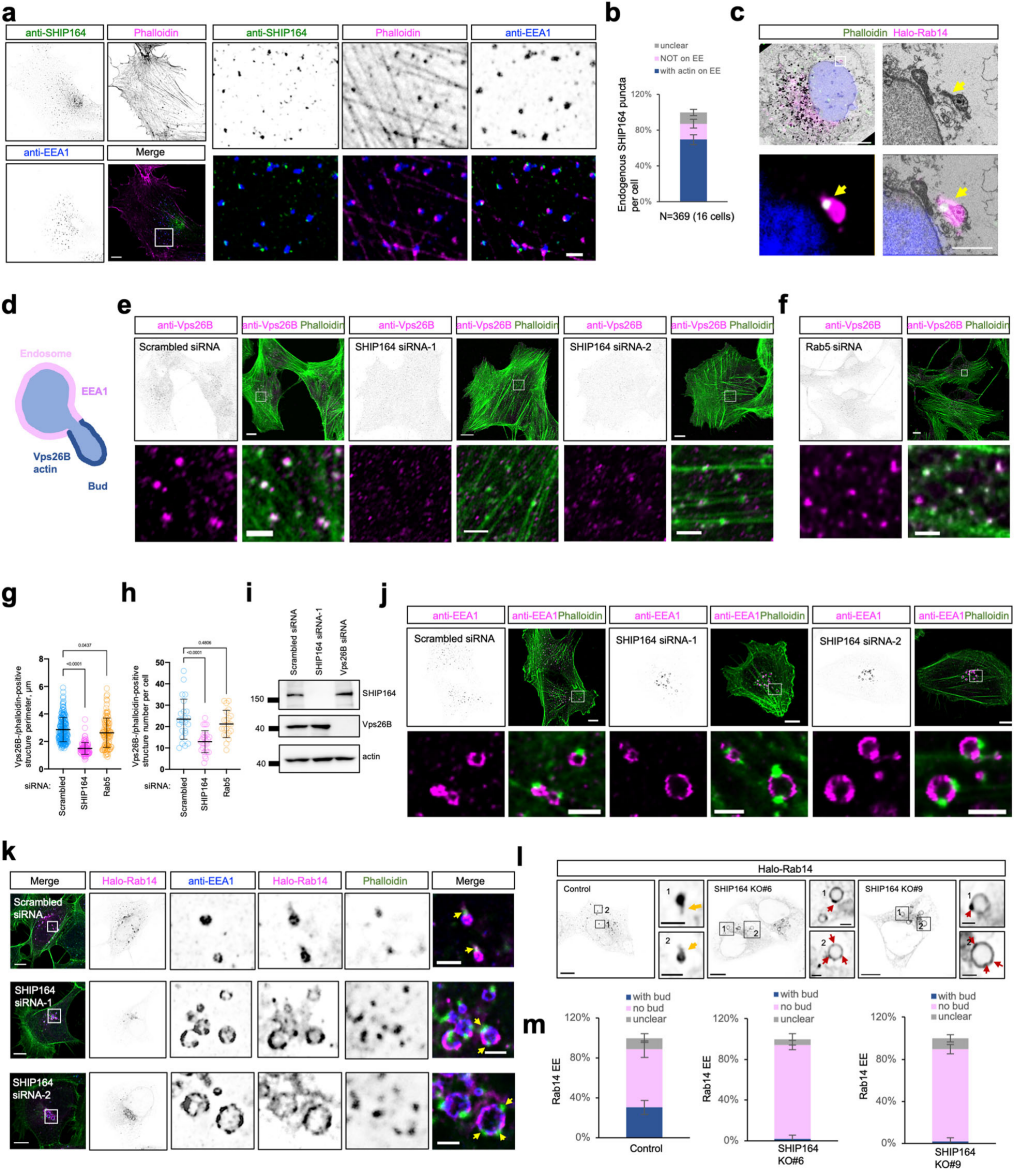
SHIP164 is required for Rab14-positive EE budding. **a** Representative images of a fixed HEK293 cell stained with pre-cleared SHIP164 antibody (green), EEA1 antibody (blue), and phalloidin (magenta). An inset was shown on the right. **b** The distribution of endogenous SHIP164 puncta. 369 SHIP164 puncta from 16 cells. Data are represented as mean ± SD. **c** CLEM of a fixed HeLa cell transfected with Halo-Rab14 (magenta) and stained with phalloidin (green) and DAPI (blue) with an inset. Arrows indicate the base of RAB14-labeled endosomal bud marked by actin foci. **d** Schematic cartoon showing two EE structures, EE vacuole, and EE bud. **e**, **f** Representative images of fixed HEK293 cells probed with antibodies against endogenous Vps26B (magenta) and phalloidin (green) upon scrambled (**e**, left), *SHIP164* siRNA (**e**, right) or *Rab5* siRNA (**f**) with insets on the bottom. **g**, **h** The size (**g**) and number (**h**) of endosome buds are positive for both Vps26B and actin in cells. More than 20 cells were quantified for each condition from three independent experiments. Ordinary one-way ANOVA with Tukey’s multiple comparisons test. Data are represented as mean ± SD. **i** Immunoblots showing that SHIP164 depletion did not affect the level of Vps26B, or vice versa. **j** Representative images of fixed HEK293 cells probed with antibodies against endogenous EEA1 (magenta) and phalloidin (green) upon scrambled or *SHIP164* siRNA with insets on the bottom. **k** Representative images of fixed HEK293 cells expressing Halo-Rab14 (magenta) and stained with antibodies against endogenous EEA1 (blue) and phalloidin (green) with insets and line-scan analyses on the right. **l** Representative images of two *SHIP164* KO HEK293 clones expressing Halo-Rab14 with two insets on the right. Yellow arrows indicated Rab14 buds in control cells and red arrows denoted Rab14 enriched foci on EE in *SHIP164* KO cells. **m** The percentage of Rab14 endosomes with buds in **l**. 1371 Rab14 EEs from 18 control cells, 478 Rab14 EEs from 18 *SHIP164* KO #6, or 583 Rab14 EEs from 20 *SHIP164* KO #9 cells were quantified from three independent experiments. Data are represented as mean ± SD. Scale bars: 10 μm in the whole cell images and 2 μm in the insets (**a**, **e**, **f**, **j**–**l**); 10 μm in the whole cell images and 1 μm in the insets (**c**).

Interestingly, we found that endogenous, untagged SHIP164 formed puncta in the cytosol that were closely associated with actin filament foci (phalloidin positive) on microdomains of EEs in HEK293 cells (Fig. 7a, b; Supplementary Fig. S7a). The specificity of SHIP164 or EEA1 antibody in IF was confirmed by *SHIP164* KO (Supplementary Fig. S7b–e) or siRNA-mediated EEA1 depletion (Supplementary Fig. S7f), respectively. The association between endogenous SHIP164 and actin foci was also observed in other cell types, including HeLa and RPE1 (Supplementary Fig. S7g).

Actin polymerization on specific endosomal microdomains was recognized as an important step for cargo sorting, membrane remodeling, and endosome fission^51,52,58–63^. Therefore, these EE microdomains, in which SHIP164 was tightly associated with actin foci, may represent EE active sites for cargo sorting (i.e., endosomal buds). Indeed, correlative light electron microscopy (CLEM) showed that actin filament foci marked the bud base of a vesicular structure, presumably an endosomal bud (Fig. 7c), consistent with a recent study^58^. Therefore, we sought to investigate whether SHIP164 was necessary for endosomal bud formation.

We examined the morphology of endosomal buds (Fig. 7d), marked by endogenous Vps26B and actin using IF staining. Indeed, endogenous Vps26B foci were well colocalized with actin foci, and depletion of SHIP164, but not Rab5, reduced the size and number of structures both positive for Vps26B and actin, as shown by high-resolution airyscan microscopy (Fig. 7e–h). This suggested a possible role of SHIP164 in the formation of EE buds. In addition, the effect of SHIP164 depletion on EE buds was not due to the decreased level of Vps26B, as immunoblot assays showed a similar level of Vps26B between control and SHIP164-depleted cells (Fig. 7i).

We next assessed the spatial relationship between EE vacuoles (anti-EEA1) and actin. Notably, in contrast to Vps26B, EEA1 decorated the EE vacuoles, but not the buds (Fig. 7j). In the control, each sorting-active EE typically had one actin foci, representing one bud base on one EE (Fig. 7j). Remarkably, SHIP164 depletion resulted in enlarged EEs with two or more actin foci (Fig. 7j). Consistently, these enlarged EEs had two or more Coronin1C foci, a regulator of actin polymerization at the base of endosomal buds^58^, in SHIP164-depleted cells (Supplementary Fig. S7h, i).

The Vps26B/actin foci marked the base of EE buds other than the whole membrane structure of buds (Fig. 7c–e)^58^. Therefore, to confirm the role of SHIP164 in EE bud formation, we examined EE buds using Rab14 as a marker. Unlike Vps26B/actin, Rab14 was localized to both vacuoles and buds (Fig. 7k; top). Remarkably, the Rab14-labeled buds on these enlarged EEs in SHIP164-depleted cells had completely disappeared, while actin foci were still present on the EEs (Fig. 7k; middle and bottom). This further supported the role of SHIP164 in EE bud formation, and suggested that SHIP164 functioned downstream of actin polymerization in Rab14 bud formation.

In addition, we confirmed the role of SHIP164 in Rab14 bud formation in *SHIP164* KO HEK293 cells. In contrast to control cells, in which a significant portion (approximately 30%) of Rab14 EEs had readily resolvable bud structures (Fig. 7l; yellow arrows), these Rab14-labeled EE buds could hardly be identified in two independent *SHIP164* KO clones. Noteworthy, Rab14 was enriched at certain microdomains of enlarged EEs in *SHIP164* KO (Fig. 7l, m; red arrows). Moreover, we found that the Rab14 bud defect was not accumulative in cells with double depletions of SHIP164 and either Vps26B or RhoBTB3 compared to the single KOs (Supplementary Fig. S8a, b), suggesting these three proteins function in the same pathway during Rab14 bud formation.

### The role of SHIP164 in Rab14 bud growth depends on its lipid transfer activity

The defect in Rab14 EE bud formation was specific to SHIP164, as the introduction of WT SHIP164 almost completely rescued the phenotype (Supplementary Fig. S8c). We next investigated whether and to what extent EE bud formation was dependent on the lipid transfer activity of SHIP164. We constructed two lipid transfer-deficient mutants. In the first mutant (mut-1), a superfolder GFP (sfGFP) was inserted into the hydrophobic groove (at residue 500) with GSSGSS linkers at either side of sfGFP, which could theoretically impede the movement of lipids through the groove (Supplementary Fig. S8d). The insertion of GFP into the hydrophobic groove could interfere with the folding of the protein, so we constructed the second mutant (mut-2), in which some hydrophobic residues in the midway of the hydrophobic groove of SHIP164 were mutated to hydrophilic residues, which were predicted to block lipid transport according to a recent study on Vps13 (Supplementary Fig. S8e)^14^. Importantly, the two potential lipid transfer-deficient mutants failed to rescue the defect in EE bud formation resulting from SHIP164 depletion (Supplementary Fig. S8f–i). The striking difference between WT and the lipid transfer-deficient mutants in the rescue experiments was not due to their expression levels, because immunoblots showed similar levels of these two proteins (Supplementary Fig. S8j). These findings indicated that lipid transfer activity of SHIP164 was required for EE bud formation.

## Discussion

In this study, we identified that Vps13-related lipid transporter SHIP164, an ATPase RhoBTB3, and a retromer subunit Vps26B formed a protein complex, which was indispensable for the biogenesis of EE buds at Golgi–EE contacts. We further demonstrated that Vps26B was a new Rab14 effector and Rab14 activity modulated the association of SHIP164 with EEs. Suppression of RhoBTB3 or Vps26B phenocopied the SHIP164 depletion. The lipid transfer activity of SHIP164 was required for EE budding. Cumulatively, we proposed a working model. In this model, active Rab14 recruits Vps26B to potential budding sites on EEs, followed by SHIP164 recruitment. When EEs form dynamic contacts with the *trans*-Golgi, SHIP164, Vps26B, and RhoBTB3 coordinate to form a dynamic protein complex at the contact sites. At such sites, SHIP164 recycles phospholipids to modulate the lipid composition of EE or Golgi membranes, and thereby promoting the formation of EE buds, and ultimately ensuring cargo sorting and transport (working model; Supplementary Fig. S9).

Recent studies have revealed a crucial role of bridge lipid transporters in organelle biogenesis. Our study extended the biological functions of bridge lipid transporters, and suggested the regulation of EE bud formation as a potential cellular function of the recently identified bridge lipid transporter SHIP164. These findings linked the lipid transfer at MCSs to cargo sorting during vesicular trafficking. SHIP164 has been shown to control the endosome to Golgi trafficking of certain cargoes, including cation-independent mannose-6 phosphate receptor (CI-MPR)^33^. Given that the retrieval pathway of unoccupied CI-MPR is dependent on the retromer complex on endosome buds^42,55,64^, the reported role of SHIP164 in the retrograde transport of CI-MPR was in line with the function of SHIP164 in endosome bud formation outlined in this study.

Notably, yeast Vps13 is recruited to multiple MCSs via different adapters on different organelles^65^. In mammals, Vps13A is recruited to ER–mitochondrial MCSs using unknown mechanisms, and it can also be recruited to ER–PM MCSs through a specific interaction with a PM-resident scramblase XK^22,66–69^. This suggests that the localizations of Vps13 proteins, including SHIP164, can be versatile and may be regulated depending on the functional states of the cells. In addition to RhoBTB3 and Vps26B identified in this study, SHIP164 also interacts with Syntaxin 6^32^ and Rab45^33^. Therefore, SHIP164 may be recruited to multiple contacts under physiological or pathological conditions.

The ER is the major site for lipid synthesis^34^. Newly synthesized lipids at the ER are then transferred to other organelles using vesicular and nonvesicular transport pathways. Therefore, various lipid transporters function at ER-associated MCSs. The ER forms contact with endosomal buds via at least two distinct tethering complexes^56,57^. The ER is tethered to endosomal budding sites via interactions between the ER adapter VAPA and a retromer subunit SNX2. The loss of VAPs results in an aberrant accumulation of PI4P at endosomal buds, further leading to actin comets at these sites, supporting the role of the ER in the control of EE budding dynamics^57^. The ER can also form contacts with endosomal buds through TMCC1–Coronin 1C interaction after the cargo has been properly segregated into the bud, followed by the formation of endosomal tubules and fission^56^. While SHIP164 has been speculated to be associated with the ER^33^, our results showed that SHIP164 was not recruited to the ER through VAPs, suggesting that SHIP164-mediated lipid transfer may not strictly rely on the ER. Indeed, we observed a longer duration of RhoBTB3-positive Golgi vesicles contacting EEs than the ER (Fig. 1m–p). In addition, RhoBTB3 depletion phenocopied the *SHIP164* KO. These findings suggest a crucial role of RhoBTB3-positive Golgi vesicles in EE bud formation.

In this study, it is still unclear how the lipid transfer of SHIP164 is linked to EE bud formation. SHIP164 may transfer glycerophospholipids to EEs for membrane expansion during EE budding. According to our results, we argued that this possibility is less likely because the Golgi might not be an optimal lipid donor organelle for glycerophospholipids. Another possibility is that SHIP164 might recycle lipids between Rab14 EE and *trans*-Golgi network to sort proteins in the absence of the ER, without a great number of net lipid traffic. This possibility is aligned with the working model of SHIP164 at Golgi–EE MCSs demonstrated in this study. In addition, the fusion of small endocytic vesicles is proposed to govern the biogenesis of endosome^70^. Given that SHIP164 localizes to clusters of small endocytic vesicles^33^, whether the fusion of these small SHIP164 vesicles to EEs also directly contributes to the membrane expansion of endosomes and/or endosomal buds is unclear. Our results support this notion by revealing a functional relationship between SHIP164 and Rab5, and its effector EEA1, two essential factors in EE fusion (Supplementary Fig. S5b–g). Importantly, RhoBTB3 is an ATPase, which may be involved in the step of vesicle uncoating before fusion during EE bud formation. In this scenario, SHIP164 may recycle a small amount of lipids to modulate the lipid compositions of EE or Golgi membranes, priming EE fusion events. While the link between the lipid transfer of SHIP164 and EE budding requires further investigation, it is clear that SHIP164-mediated lipid transfer and its interacting partners RhoBTB3 and Vps26B are essential for this fundamental process.

Because SHIP164 is highly expressed in the brain, and recent clinical studies suggest its role in Parkinson’s disease^71^ and myopia, or nearsightedness, a common ocular genetic disorder^72^, our results will provide new mechanistic insights into these syndromes.

## Materials and methods

### Cell culture, transfection, RNAi

HEK293 cells (ATCC) and human cervical cancer HeLa cells (ATCC) were grown in DMEM (Invitrogen) supplemented with 10% fetal bovine serum (FBS, Gibco) and 1% penicillin/streptomycin (Invitrogen). The hTERT-immortalized retinal pigment epithelial cell line hTERT RPE-1 cell (ATCC) was grown in DMEM/F12 (Gibco) supplemented with 10% FBS and 1% penicillin/streptomycin. All of the cell lines used in this study were confirmed free of mycoplasma contamination.

Transfection of plasmids and RNAi oligos was carried out with Lipofectamine 2000 (Invitrogen) and RNAi MAX (Invitrogen), respectively. For transfection, cells were seeded at 4 × 10^5^ cells per well in a 6-well dish ~16 h before transfection. Plasmid transfections were performed in OPTI-MEM (Invitrogen) with 2 μL Lipofectamine 2000 per well for 6 h, followed by trypsinizing and replating cells onto glass-bottom confocal dishes at ~3.5 × 10^5^ cells per well. Cells were imaged in live-cell medium (DMEM with 10% FBS and 20 mM HEPES without penicillin or streptomycin) ~16–24 h after transfection. For all transfection experiments in this study, the following amounts of DNA were used per 3.5 cm well (individually or combined for co-transfection): 1000 ng for GFP-SHIP164 and its mutants; 500 ng for Halo-RhoBTB3 and its mutants; OFP-VPS26B; 500 ng for BFP-Golgi; OFP-Rab5A. For siRNA transfections, cells were plated on 3.5 cm dishes at 30%–40% density, and 2 μL Lipofectamine RNAimax and 50 ng siRNA were used per well. At 48 h after transfection, a second round of transfection was performed with 50 ng siRNAs. Cells were analyzed 24 h after the second transfection for suppression.

### *SHIP164* KO HEK293 cell lines

To make *SHIP164* KO cell lines, two gRNAs (5′-GTACACAGCCCAAACATCCG-3′ and 5′-AGGTCAGAATGTCGACAGTT-3′) were used to delete 223 bp from the exon 14 of *SHIP164* gene (Supplementary Fig. S7b). Complementary gRNAs were annealed and subcloned into the pSpCas9(BB)-2A-GFP (pX-458) vector (48138, Addgene) between *Bbs*I endonuclease restriction sites. Upon transfection, single cells containing GFP fluorescence signals were sorted using FACS into 96-well plates, and the growing clones were verified by PCR. The existence of mutations was confirmed by Sanger sequencing. The mutations led to code shifting and early termination in the SHIP164 coding sequence, and the expression of protein level was verified by western blot analysis (Supplementary Fig. S7d). Two validated clones (#6 and #9) were used.

### Antibody clearing

SHIP164 antibody was precleared with *SHIP164* KO HEK293 lysates to improve signal to noise. *SHIP164* KO HEK293 cells were fixed in 4% PFA for 20 min at room temperature, washed twice with PBS, permeabilized with 0.1% Triton X-100 in PBS (PBX) for 5 min and scraped in PBX with 1% Triton X-100. SHIP164 antibody was added to the fixed cells, mixed for 12 h at 4 °C with gentle rocking. The supernatant containing cleared antibodies was collected by centrifugation at 17,000× *g* for 20 min at 4 °C, and then used for IF staining.

### GFP-trap assay

GFP trap was used for the detection of protein–protein interactions and the GFP-Trap assays were performed according to the manufacturer’s protocol. 5% input was used in GFP traps unless otherwise indicated. Briefly, cells were lysed in Lysis buffer (50 mM Tris-Cl pH 7.5, 150 mM NaCl, 0.5 mM EDTA, 0.5% Nonidet^TM^ P40 Substitute). Lysates were centrifuged at 20,000× *g* for 10 min at 4 °C and remove the pellets. Supernatants were incubated with GFP-Trap agarose beads for 3 h at 4 °C with gentle rocking. The beads were pelleted and washed three times with wash buffer (50 mM Tris-HCl, pH 7.5, 150 mM NaCl, 0.5 mM EDTA, 1× Proteases Inhibitor cocktail), and then boiled with SDS sample buffer. Proteins of interest were analyzed by immunoblotting. 5% input was used in GFP traps unless otherwise indicated.

### Endogenous Immunoprecipitation

Cells were lysed, followed by incubation with 1 μg antibody for 8 h at 4 °C with gentle rocking. IgG was used as a negative control. Then, the mixture was incubated with protein A/G beads for another 12 h. The beads were washed three times, and then boiled with SDS sample buffer.

### IF staining

Cells were fixed with 4% paraformaldehyde (PFA, Sigma) in PBS for 20 min at room temperature. After washed with PBS three times, cells were permeabilized with 0.5% Triton X-100 in PBS for 10 min on ice. Cells were washed three times with PBS, and blocked with 3% BSA in PBS for 1 h, followed by incubation with primary antibodies in diluted blocking buffer overnight. The samples were washed with PBS for three times. Secondary antibodies were applied for 1 h at room temperature. After washing with PBS three times, the samples were mounted on Vectashield (H-1000; Vector Laboratories).

### Live imaging by high-resolution confocal microscopy

Cells were grown on 35 mm glass bottom confocal MatTek dishes, and the dishes were loaded to a laser scanning confocal microscope (LSM900, Zeiss) equipped with multiple excitation lasers (405 nm, 458 nm, 488 nm, 514 nm, 561 nm, and 633 nm) and a spectral fluorescence GaAsP array detector. Cells were imaged with the 63×/1.4 NA iPlan-Apochromat 63× oil objective using the 405 nm laser for BFP, 488 nm for GFP, 561 nm for OFP, tagRFP or mCherry and 633 nm for Janilia Fluo^®^ 646 HaloTag^®^ Ligand.

### Protein expression and purification

His-tag or GST-tag construct was transformed into TSsetta (DE3) chemically competent cells (TSC04, Tsingke). Cells were incubated at 37 °C until the OD_600_ reached 0.6–0.8. Followed by induction with 1 mM IPTG overnight at 16 °C, cells were lysed via sonication. Cell lysates were centrifuged at 14,000× *g* for 30 min. The supernatant was incubated with Ni-NTA resin (G600033-0100, Sangon, for His-fusion protein) or GST-tag Resin (C600031-0025, Sangon, for GST-fusion protein), and the resins were passed through via gravity flow.

### MS identification of GST-SHIP164-CT-interacting proteins

The identification of GST-SHIP164-CT-interacting proteins by MS was described in our previous study^20^. Briefly, GST constructs were transformed into *Escherichia coli* BL21 (DE3) cells, which were then incubated at 37 °C until the OD_600_ reached 0.6–0.8. After that, protein expression was induced with 0.5 mM IPTG, and then cells were cultured at 16 °C for another 16 h. Cells were pelleted, resuspended in buffer A (20 mM Tris-HCl, pH 7.5, 300 mM NaCl, 1 mM DTT, and 10% glycerol) supplemented with protease inhibitors (Topscience) and lysed via sonication. GST fusion proteins were purified via the GST-tag Protein Purification kit (C600031-0025, Sangon). Protein digestion was performed with FASP method. Briefly, the detergent, DTT and IAA in UA buffer was added to block-reduced cysteine. Finally, the protein suspension was digested with 2 µg trypsin (Promega) overnight at 37 °C. The peptide was collected by centrifugation at 16,000× *g* for 15 min. The peptide was desalted with C18 StageTip for further LC-MS analysis. LC-MS/MS experiments were performed on a Q Exactive Plus mass spectrometer that was coupled to an Easy nLC (ThermoFisher Scientific). Peptide was first loaded to a trap column (100 µm × 20 mm, 5 µm, C18, Dr Maisch GmbH, Ammerbuch) in buffer A (0.1% formic acid in water). Reverse-phase high-performance liquid chromatography (RP-HPLC) separation was performed using a self-packed column (75 µm × 150 mm; 3 µm ReproSil-Pur C18 beads, 120 Å, Dr Maisch GmbH, Ammerbuch,) at a flow rate of 300 nL/min. The RP-HPLC mobile phase A was 0.1% formic acid in water, and B was 0.1% formic acid in 95% acetonitrile. The gradient was set as follows: 2%–4% buffer B from 0 to 2 min, 4%–30% buffer B from 2 to 47 min, 30%–45% buffer B from 47–52 min, 45%–90% buffer B from 52 to 54 min, and 90% buffer B kept until to 60 min. MS data was acquired using a data-dependent top 20 method dynamically choosing the most abundant precursor ions from the survey scan (350–1800 m/z) for HCD fragmentation. A lock mass of 445.120025 Da was used as an internal standard for mass calibration. The full MS scans were acquired at a resolution of 70,000 at m/z 200, and 17,500 at m/z 200 for MS/MS scan. The maximum injection time was set to 50 ms for MS and 50 ms for MS/MS. Normalized collision energy was 27 and the isolation window was set to 1.6 Th. Dynamic exclusion duration was 60 s. The MS data were analyzed using MaxQuant software version 1.6.1.0. MS data were searched against the UniProtKB Human norvegicus database (36,080 total entries, downloaded on 08/14/2018). Trypsin was selected as the digestion enzyme. A maximum of two missed cleavage sites and a mass tolerance of 4.5 ppm for precursor ions and 20 ppm for fragment ions were defined for database search. Carbamidomethylation of cysteines was defined as a fixed modification, while acetylation of protein N-terminal, oxidation of Methionine was set as variable modifications for database searching. The database search results were filtered and exported with a < 1% false discovery rate at peptide-spectrum-matched level, and protein level, respectively.

### GST-pull-down assays

The purified GST protein was incubated with GST tag Resin at 4 °C for 12 h, washed ten times with HNM buffer (20 mM HEPES, pH 7.4, 100 mM NaCl, 5 mM MgCl_2_, 1 mM DTT, 0.2% NP-40) and centrifuged at 1000× *g* for 2 min to remove supernatant. Corresponding His fusion protein was then added, incubate at 4 °C for another 12 h, washed with HNM buffer, and boiled with SDS sample buffer. Western blotting was performed using anti-GST or His antibodies.

### GFP-pull-down assays

HEK293 cells transiently transfected with GFP-Rab14 were lysed in high-salt lysis buffer (RIPA buffer containing 500 mM NaCl, proteasome inhibitors, and PMSF). GFP-Trap beads were used to pellet GFP-Rab14 from cell lysates, followed by washing with high-salt lysis buffer ten times. The GFP-Rab14 beads were incubated with purified GST-SHIP164-CT overnight at 4 °C, washed with freshly prepared HNM buffer, and boiled with SDS sample buffer.

### Duolink PLA fluorescence protocol

HEK293 cells were fixed with 4% PFA in PBS for 20 min at room temperature. After washing with PBS for three times, cells were permeabilized with 0.5% Triton X-100 in PBS for 10 min on ice. Cells were then washed three times with PBS, and blocked with Duolink Blocking Solution for 1 h at 37 °C. Cells were incubated in the Duolink Antibody Diluent with diluted primary antibodies overnight at 4 °C. The cells were washed 2 times with 5 min for each wash in 1× Wash Buffer A at room temperature and the PLA probe solution was applied for 1 h at 37 °C. The cells were washed 2 times with 5 min each in 1× Wash Buffer A at room temperature and then incubated in the ligation solution for 30 min at 37 °C. The cells were washed 2 times with 5 min each in 1× Wash Buffer A at room temperature and were added to the amplification solution for 100 min at 37 °C, followed by 2 wash with 10 min each in 1× Wash Buffer B at room temperature. Eventually, the samples were washed in 0.01× Wash Buffer B for 1 min, followed by incubation in a mounting medium with DAPI.

### CLEM

For CLEM, the procedures were described in our previous study^21^. Briefly, cells were grown on glass-bottom P35G-2-14-C-Grid dishes (MatTek). The dishes have a high optical quality coverslip with a photo-etched grid and coordinate to facilitate pinpointing the location of individual cells. The cells were fixed with 4% PFA (Ted Pella Co., Ltd.) in 0.1 M PB buffer (pH 7.0) for 30 min at room temperature. Once the cells of interest were found, their positions on the grid were documented by switching from fluorescence to differential interference contrast mode. After fluorescence imaging, the selected areas with positive cells were marked on the bottom of the coverslip under a light microscope to facilitate the processing of EM. After observation with a confocal microscope (Zeiss LSM 980), the samples were fixed in 2.5% glutaraldehyde (Ted Pella Co., Ltd.) and 2% PFA mixture in 0.1 M PB buffer (pH 7.0) at 4 °C overnight. After washed in PB buffer three times, the samples were stained with 2% OsO_4_ and 1.5% potassium hexacyanoferrate, and sequentially washed with PB buffer (three times) and ddH_2_O (three times). Then, the samples were incubated in 1% TCh and washed with ddH_2_O four times, stained in 2% OsO_4_, and wahed with ddH_2_O four times. The samples were then stained with 1% UA, and then washed with ddH_2_O 4 times. The samples were stained with a lead aspartate solution and washed with ddH_2_O five times. Then, the samples were dehydrated by incubating with ethanol (30, 50, 70, 95, and 100% twice), followed by incubation with acetone twice. After hydration, the samples were subjected to infiltration and embedding step, in which samples were sequentially infiltrated with acetone/Epon resin mixtures with three ratios (acetone:Epon = 3:1; 1:1; 1:3) and 100% Epon three times, followed by polymerization with Epon for 48 h at 60 °C. Eventually, the samples were subjected to a serial ultrathin sections, and the sections were observed at 80 kV in an FEI Talos 120 kV transmission electron microscopy (ThermoFisher Scientific).

### Statistical analysis

All statistical analyses and *P* value determinations were performed in GraphPad Prism6. All the error bars represent mean ± SD. To determine values, ordinary one-way ANOVA with Tukey’s multiple comparisons test was performed among multiple groups, and a two-tailed unpaired Student’s *t*-test was performed between two groups.

### Image analysis

All image analysis and processing were performed using ImageJ (2.0.0-rc-66/1.52b, NIH). Colocalization-based analysis of Golgi–EEs MCSs was performed using a plugin named colocalization in ImageJ with the following settings: Ratio (0–100%): 50; threshold channel 1 (0–255):50; threshold channel 2 (0–255):50; display value (0–255):255. MCSs were automatically identified by the colocalization plugin, with white pixels representing potential MCSs.

### Supplementary information

Supplementary Information

Vdieo-1

Vdieo-2

Vdieo-3

## Data Availability

All the data and relevant materials, including reagents and primers, that support the findings of this study are available from the corresponding author upon reasonable request.
