# Supplementary material for: Biogenesis of Rab14-positive endosome buds at Golgi–endosome contacts by the RhoBTB3–SHIP164–Vps26B complex
Source: Cell Discov. 2024 Apr 2;10:38. doi: 10.1038/s41421-024-00651-6 (PMC10987540; doi:10.1038/s41421-024-00651-6)

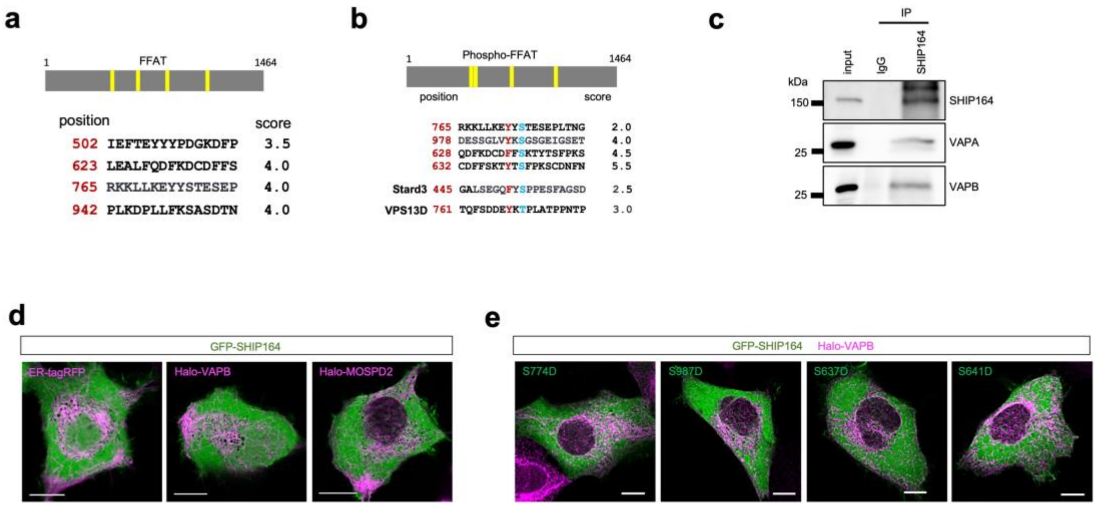

Supplementary Fig. S1 Weak interactions between SHIP164 and VAPs.
a-b FFAT motifs (**a**) or phospho-FFAT motifs (**b**) were predicted in SHIP164. **c** CoIP assays show interactions between endogenous SHIP164 and endogenous VAPA or VAPB in HEK293 cells. **d** Representative images of live HEK293 cells expressing GFP-SHIP164 (green) along with either a general ER marker ER-tagRFP (magenta; **left**), Halo-VAPA (magenta; **middle**), or Halo-Mospd2 (magenta; **right**). **e** Representative images of live HEK293 cells expressing Halo-VAPB (magenta) and GFP-SHIP164 FFAT phosphomimetic mutants including S774D, S987D, S637D, and S641D (green). Scale bar, 10μm in the whole cell images in (d-e).

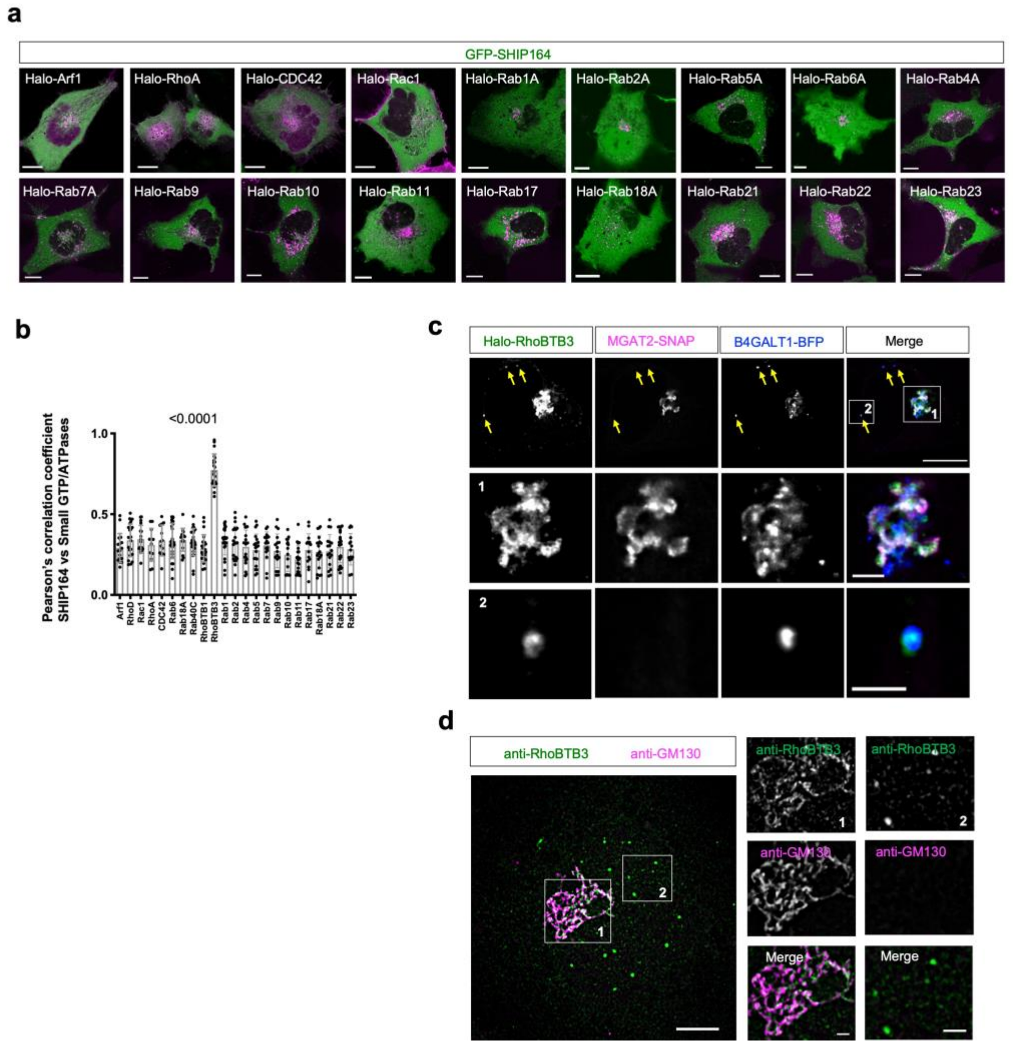

Supplementary Fig. S2 Identification of RhoBTB3 as a novel SHIP164 interacting protein on Golgi.

a Representative images of live HEK293 cells expressing GFP-SHIP164 (green) and small GTPases (magenta). **b** Pearson's correlation coefficient of GFP-SHIP164 vs Arf1(17 cells); RhoA (13 cells); CDC42 (16 cells); Rac1 (18 cells); Rab1A (26 cells); Rab2A (22 cells); Rab4A (23 cells); Rab5A (21 cells); Rab6A (26 cells); Rab7A (19 cells); Rab9 (18 cells); Rab10 (17 cells); Rab11 (23 cells); Rab17 (16 cells); Rab18A (21 cells); Rab40c (27 cells); RhoBTB1 (23 cells); RhoBTB3 (21 cells); Rab21 (19 cells); Rab22 (26 cells); and Rab23 (16 cells) in more than 3 independent experiments. Ordinary one-way ANOVA with Tukey's multiple comparisons test. Mean ± SD. **c** Representative images of a live HEK293 cell expressing Halo-RhoBTB3 (green), MGAT2-SNAP (magenta), and B4GALT1-BFP (blue). Yellow arrows denote Halo-RhoBTB3 on trans-Golgi vesicles marked by B4GALT1-BFP, but not on cis-Golgi. **d** Representative images of a fixed HEK293 cell stained with RhoBTB3 antibody (green) and anti-GM130 (magenta) with two insets on the right. Scale bar, 10μm in the whole cell images and 2μm in the insets in (a, c-d).

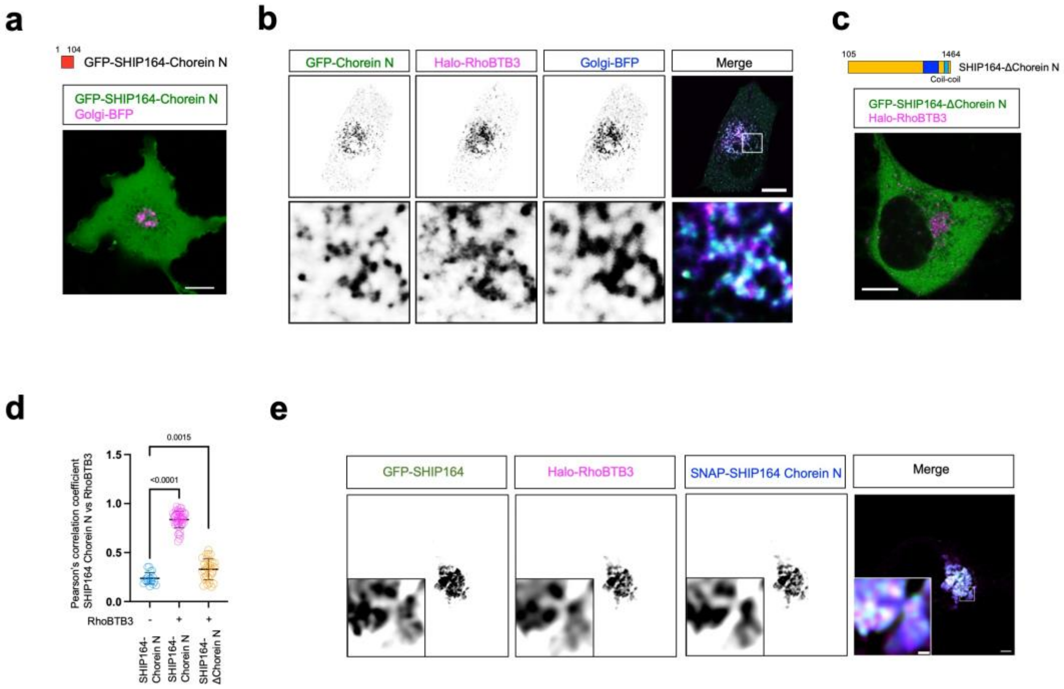

Supplementary Fig. S3 Dissection of SHIP164-RhoBTB3 interaction.
a Representative images of a live HEK293 cell expressing GFP-SHIP164-Chorein N (green) and Golgi-BFP (magenta). **b** Representative images of a live HEK293 cell expressing GFP-SHIP164-Chorein N (green), Halo-RhoBTB3 (magenta), and Golgi-BFP (blue) with insets on the bottom. **c** Representative images of a live HEK293 cell expressing GFP-SHIP164-ΔChorein N (green) and Halo-RhoBTB3 (magenta). **d** Pearson's correlation coefficient of SHIP164 proteins vs the Golgi; GFP-SHIP164-Chorein N without Halo-RhoBTB3 (18 cells), with Halo-RhoBTB3 (46 cells), or GFP-SHIP164- Δ1-104 (29 cells) in more than 3 independent experiments. Ordinary one-way ANOVA with Tukey's multiple comparisons test. Mean ± SD. **e** Representative images of a live HEK293 cell expressing GFP-SHIP164 (green), Halo-RhoBTB3 (magenta) and SNAP-SHIP164-Chorein N (blue) with insets. Scale bar, 10μm in the whole cell images and 2μm in the insets in (a-c, e).

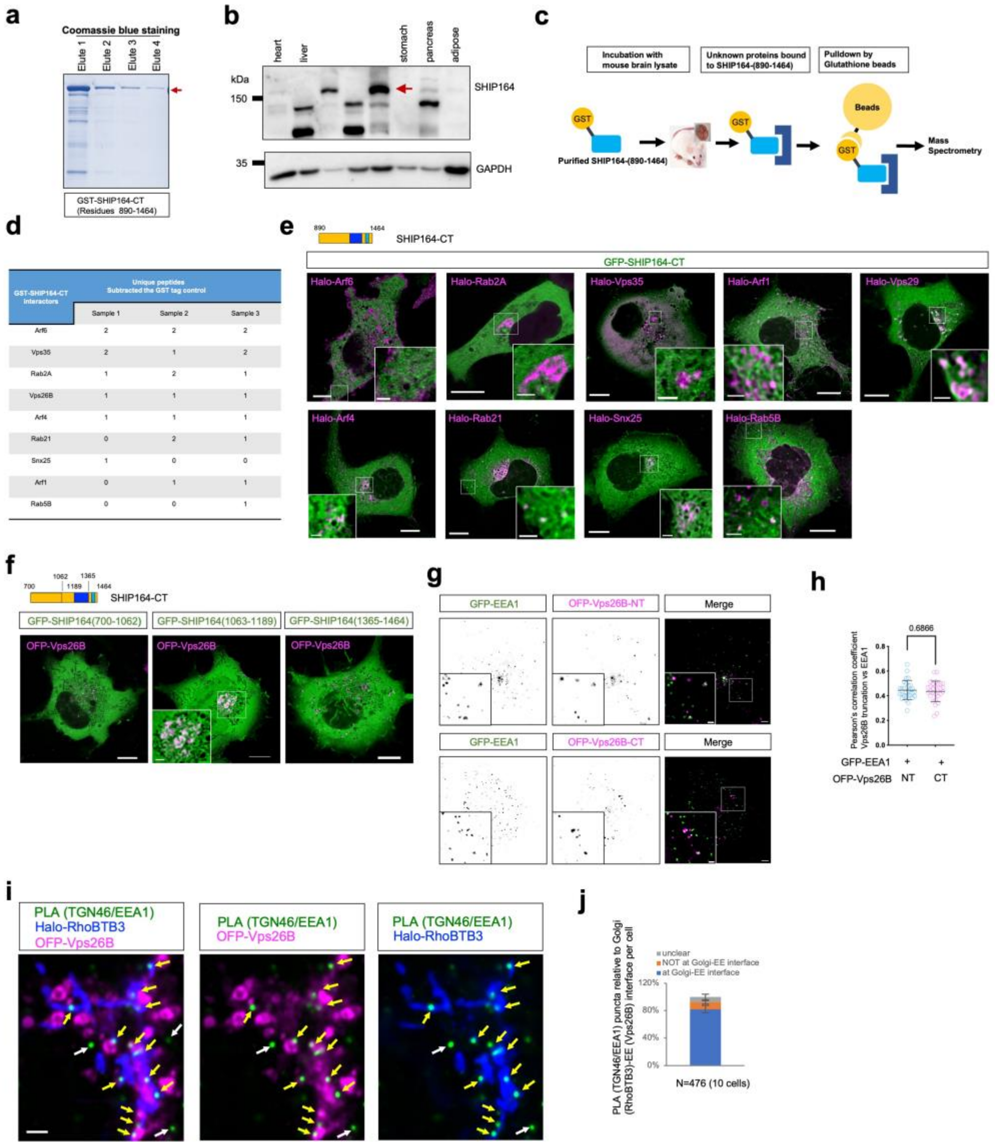

GST-SHIP164-CT interactors	Unique peptides Subtracted the GST tag control		
	Sample 1	Sample 2	Sample 3
Arf6	2	2	2
Vps35	2	1	2
Rab2A	1	2	1
Vps26B	1	1	1
Arf4	1	1	1
Rab21	0	2	1
Snx25	1	0	0
Arf1	0	1	1
Rab5B	0	0	1

Supplementary Fig. S4. Supplemental results to Fig 2 and Fig 3.
a Coomassie blue staining of purified GST-SHIP164-CT (residues 890-1464). **b** Western blots of SHIP164 in mouse tissues with red arrow denoting SHIP164. **c** Schematic cartoon of GST pulldown assays using purified SHIP164-CT in mouse brain. **d** A list of protein candidates that functionally related to the endosome-Golgi trafficking, after removal of proteins pull-downed by GST tag alone. **e** Representative images of HEK293 cells expressing GFP-SHIP164-CT (green) and either Halo-Arf6, Halo-Rab2A, Halo-Vps35, Halo-Arf1, Halo-Arf4, Halo-Rab21, Halo-Snx25, Halo-Rab5B or Halo-Vps29 (magenta) with insets. **f** Representative images of a HEK293 cell expressing either GFP-SHIP164(700-1062), GFP-SHIP164(1063-1189) or GFP-SHIP164(1365-1464), and OFP-Vps26B (magenta) with insets. **g** Representative images of a HEK293 cell expressing either OFP-Vps26B-NT (magenta, top) or OFP-Vps26B-CT (magenta, bottom) and GFP-EEA1 with insets. **h** Pearson's correlation coefficient of EEA1 vs either Vps26B-NT (29 cells) or Vps26B-CT (29 cells) in more than 3 independent experiments. Two-tailed unpaired student t-test. Mean ± SD. **i** Representative PLA images of a fixed HEK293 cell expressing OFP-Vps26B (magenta) and Halo-RhoBTB3 (blue) in HEK293 cells with insets. **j** The distribution of PLA (TGN46/EEA1) puncta relative to Golgi -EE as in (I) from 3 independent assays. (476 PLA puncta from 10 cells). Mean ± SD. Scale bar, 10μm in the

whole cell images and 2μm in the insets in (e-g, i).

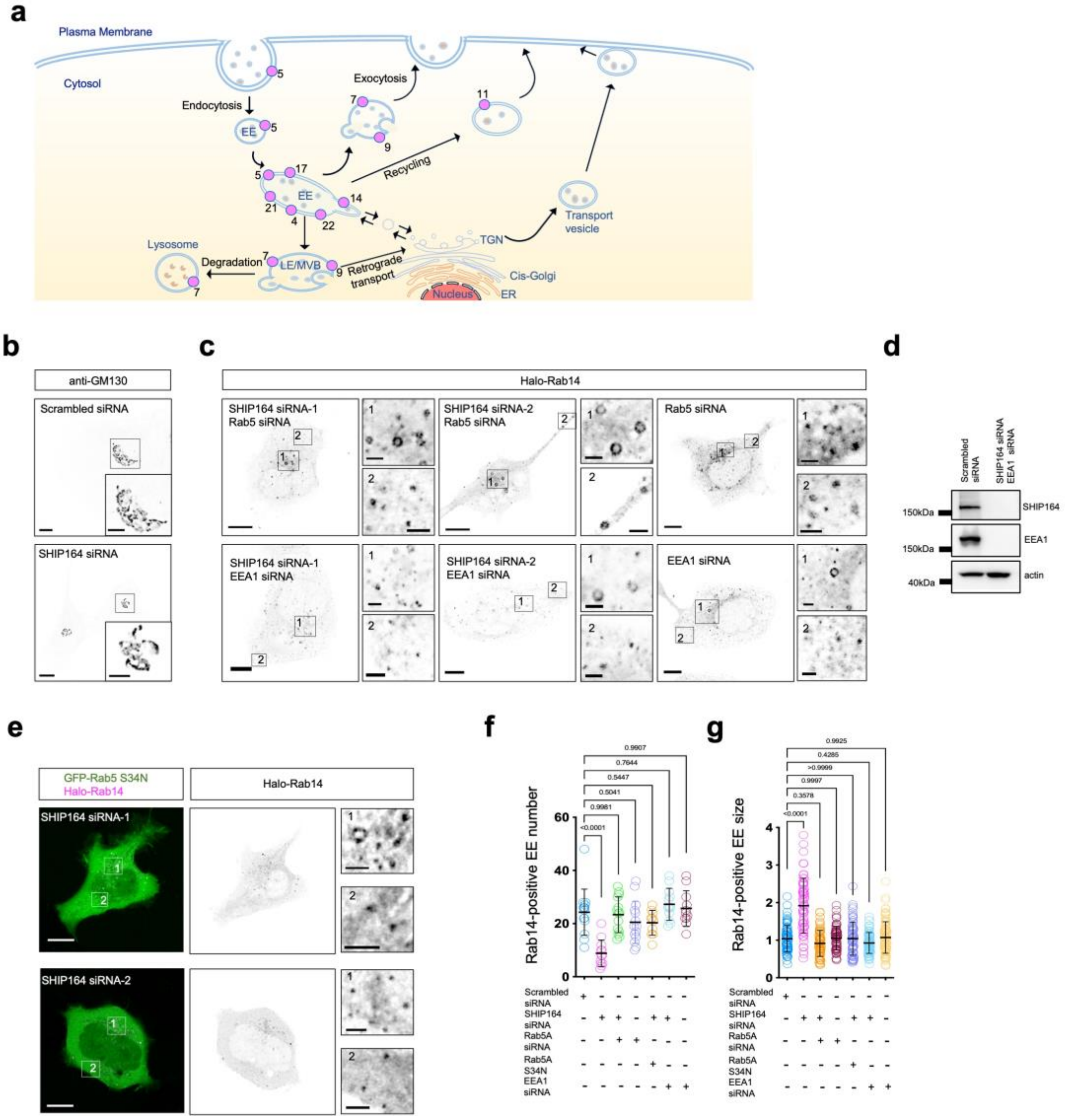

Supplementary Fig. S5. Depletion of either Rab5 or EEA1 suppresses the Rab14 defect in SHIP164-depleted cells.

a Schematic cartoon briefly showing membrane compartments of Rabs in vesicular trafficking[1]. **b** Representative images of fixed HEK293 cells stained with antibodies against endogenous GM130 upon scrambled or SHIP164 siRNAs with insets. **c** Representative images of fixed HEK293 cells expressing Halo-Rab14 upon SHIP164 and Rab5 siRNAs, SHIP164 and EEA1 siRNAs or Rab5 siRNAs with two insets. **d** Immunoblots showing the efficiency of SHIP164, EEA1 and Rab5 depletion. **e** Representative images of live HEK293 cells expressing dominant-negative Halo-Rab5 S34N (green) and Halo-Rab14 (magenta) upon SHIP164 depletion with two insets. **f-g** The number (**f**) or size (**g**) of Rab14-positive EEs per cell in scrambled, SHIP164, SHIP164 and Rab5, SHIP164 and Halo-Rab5 S34N expression, Rab5 alone, SHIP164 and EEA1, or EEA1 siRNA alone treated cells based on Fig. S5b-e. More than 20 cells from 3 independent assays were quantified for each condition. Ordinary one-way ANOVA with Tukey's multiple comparisons test. Mean ± SD. Scale bar, 10μm in the whole cell images and 2μm in the insets in (b-c, e).

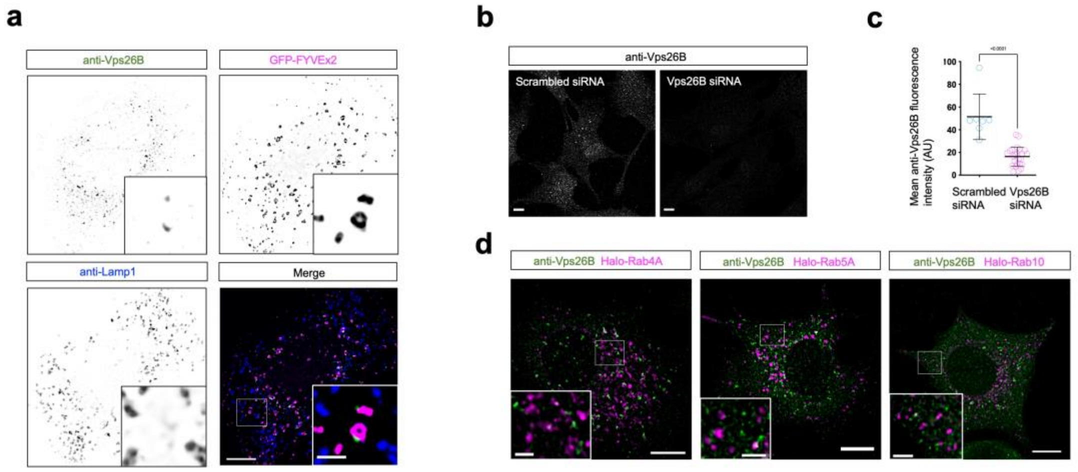

Supplementary Fig. S6 Supplemental results to Fig 5.
a Representative images of fixed HEK293 cells stained with Vps26B antibody (green) and Lamp1 antibody (blue) and expressing GFP-FYVEx2 (magenta) with insets. **b** Representative images of fixed HEK293 cells stained with Vps26B antibody (green) upon scrambled or Vps26B siRNAs treatments. **c** Mean intensity of anti-Vps26B fluorescence intensity per cell in scrambled (7 cells) or Vps26B siRNA (25 cells) treated cells. Two-tailed unpaired student t-test. Mean ± SD. **d** Representative images of fixed HEK293 cells stained with Vps26B antibody (green) and expressing Halo-Rab4A, Halo-Rab5A, or Halo-Rab10 (magenta) with insets. Scale bar, 10μm in the whole cell images and 2μm in the insets in (a-b, d).

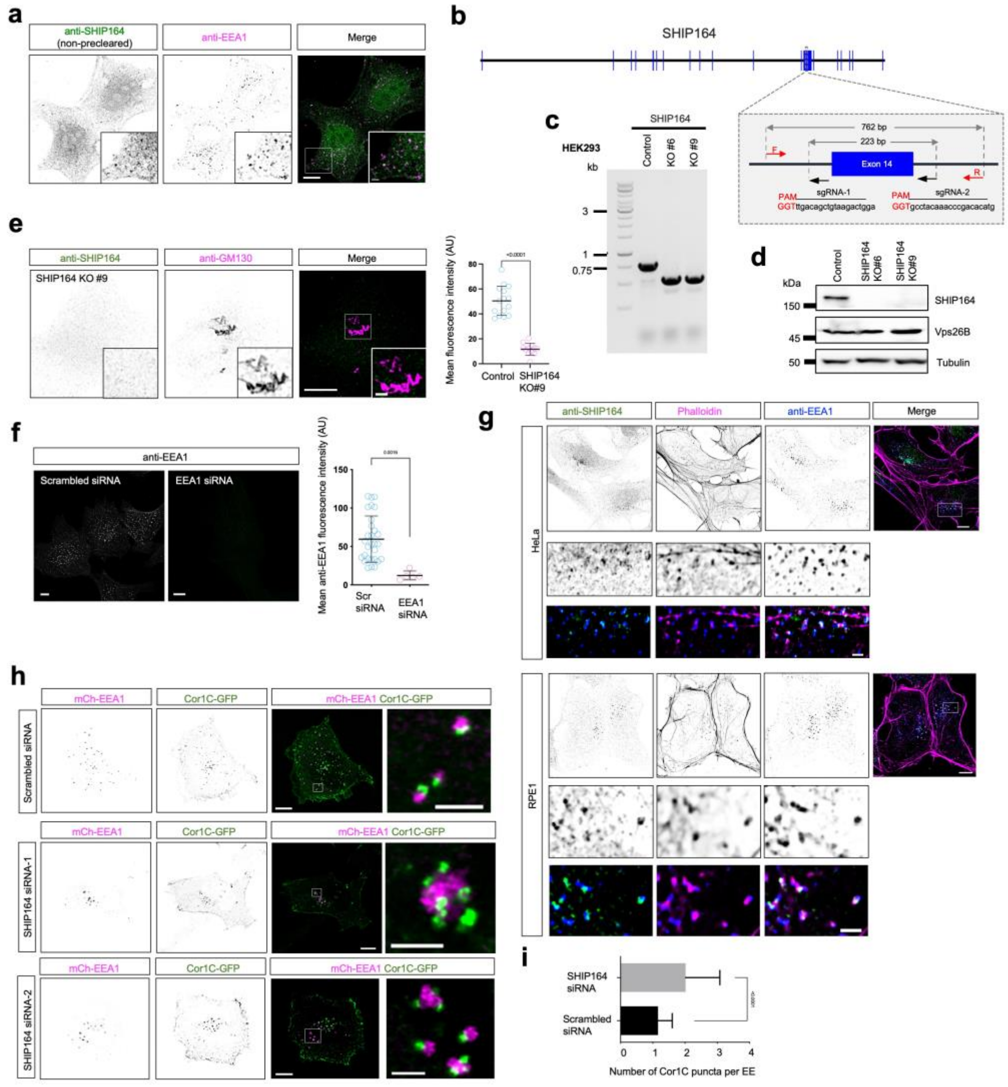

Supplementary Fig. S7 Supplemental results to Figure 7.

a Representative images of fixed HEK293 cells stained with raw SHIP164 antibody (green) and EEA1 antibody (magenta) with an inset. **b** Schematic diagram of crispr-cas9-mediated KO of SHIP164 in HEK293 cells. **c-d** Two SHIP164 clones (#6 and #9) were confirmed by DNA gels (**c**) and immunoblots (**d**). Note that Vps26B levels were not affected by SHIP164 KO. **e** Left: Representative images of fixed SHIP164 KO HEK293 cells (clone #9) stained with pre-cleared SHIP164 antibody (green) and GM130 antibody (magenta) with an inset. Right: fluorescence intensity of anti-SHIP164 in control (15 cells) or SHIP164 KO #9 (12 cells). Two-tailed unpaired student t-test. Mean ± SD. **f** Left: Representative images of fixed HEK293 cells stained with EEA1 antibody (green) upon scrambled or EEA1 siRNA treatments. Right: fluorescence intensity of anti-EEA1 in scrambled (29 cells) or EEA1 siRNA (5 cells)-treated cells. Two-tailed unpaired student t-test. Mean ± SD. **g** Representative images of fixed HeLa (**upper**) or RPE1 cells (**bottom**) stained with pre-cleared SHIP164 antibody (green), EEA1 antibody (blue) and phalloidin (magenta) with an inset on the bottom. **h** Representative images of HEK293 cells expressing mCh-EEA1 (magenta) and Cor1C-GFP (green) upon scrambled or SHIP164 siRNA with insets on the right. **i** The number of Cor1C puncta per EE in scrambled (14 cells) or SHIP164 siRNA (15 cells)-treated cells from 3 independent assays based on Fig. S8H. Two-tailed unpaired student t-test. Mean ± SD. Scale bar, 10μm in the whole cell images and 2μm in the insets in (a, e-h).

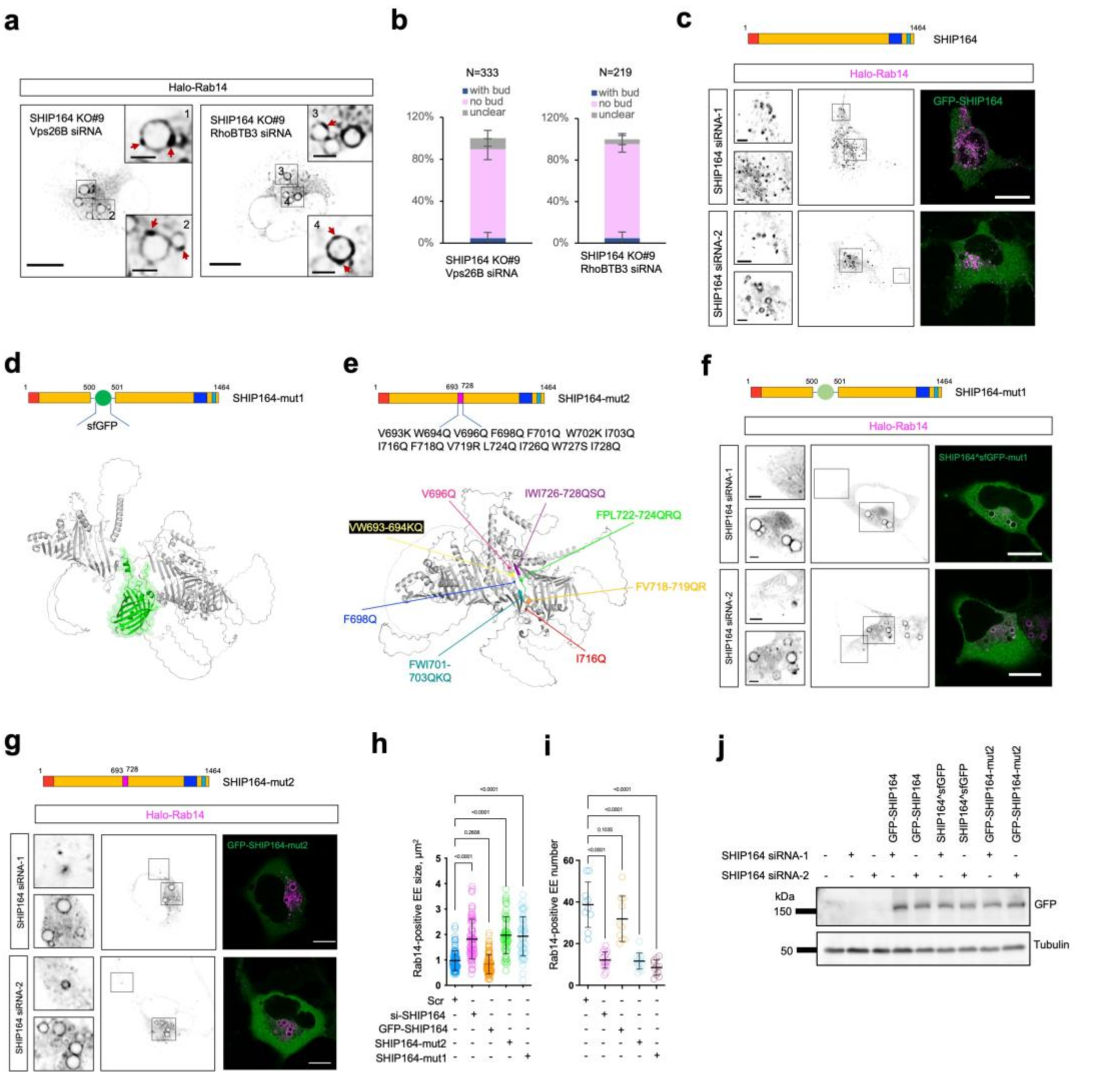

Supplementary Fig. S8 Lipid transfer of SHIP164 is required for the growth of Rab14 EE buds.

a Representative images of live SHIP164 KO HEK293 clone#9 expressing Halo-Rab14 upon siRNAs targeting Vps26B (**left**) or RhoBTB3 (**right**) with two insets. Red arrows denoted Rab14 foci on EEs. **b** The percentage of Rab14 endosomes with buds in (**a**). 333 Rab14 EEs from 18 Vps26B siRNA-treated SHIP164 KO#9 cells, 219 Rab14 EEs from 15 RhoBTB3 siRNA-treated SHIP164 KO#9 were quantified from 3 independent experiments. Mean ± SD. **c** Representative images of SHIP164-depleted HEK293 cells expressing siRNA-resistant GFP-SHIP164 along with Halo-Rab14 (magenta) with two insets on the left. **d-e** AlphaFold predicted structures of two SHIP164 lipid transfer-defective mutants: SHIP164^sfGFP (mut-1; **d**) or SHIP164-mut2 (**e**). In mut-1, sfGFP is highlighted in green; in mut-2, hydrophobic residues in the midway of hydrophobic groove are highlighted in color. **f-g** Representative images of SHIP164-depleted HEK293 cells expressing SHIP164-mut1 (**f**), or SHIP164-mut2 (**g**), along with Halo-Rab14 (magenta) with two insets on the left. **h-i** The perimeter (**h**) and number (**i**) of Rab14 endosomes in (**c, f-g**). More than 20 cells were quantified for each condition from 3 independent experiments. Ordinary one-way ANOVA with Tukey's multiple comparisons test. Mean ± SD. **j** Immunoblots showing the level of siRNA-resistant GFP-SHIP164, GFP-SHIP164-mut2, or SHIP164^sfGFP (mut1) in the rescue experiments in (**c, f-g**). Scale bar, 10μm in the whole cell images and 2μm in the insets in (a, c, f-g).

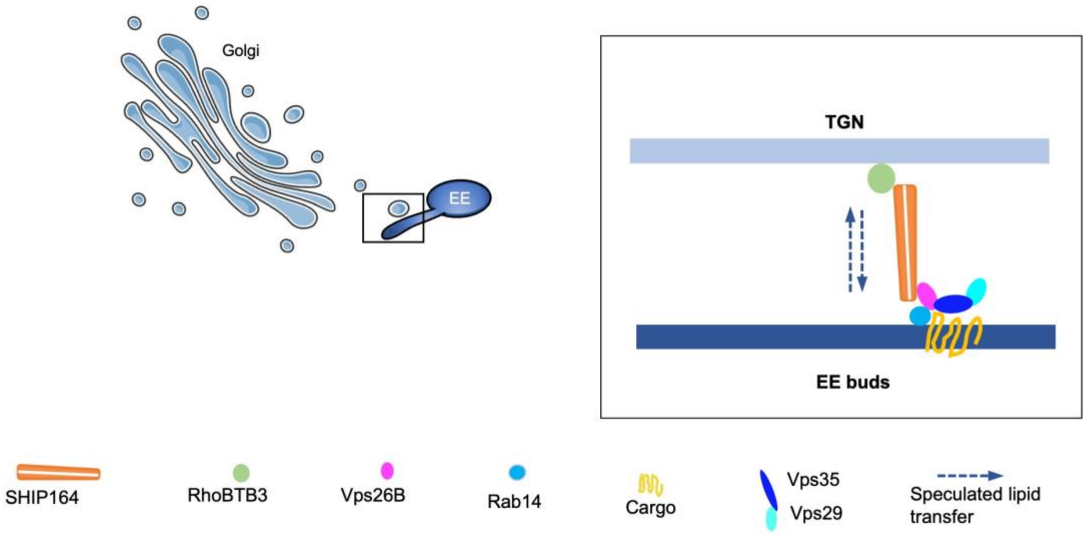

Supplementary Fig. S9 Working model of the RhoBTB3-SHIP164-Vps26B complex in the growth of Rab14 EE buds.
Vps26B is a Rab14 effector, and active Rab14 recruits Vps26B to potential EE budding sites, followed by SHIP164 recruitment. When EEs form dynamic contacts with the trans-Golgi network, SHIP164, Vps26B, and RhoBTB3 coordianted to form a dynamic protein complex at the contact sites. At such sites, SHIP164 recycles phospholipids to modulate the lipid compositions of EE or Golgi membranes, and thereby promotes the formation of EE buds, and consequently ensures cargo sorting and transport.

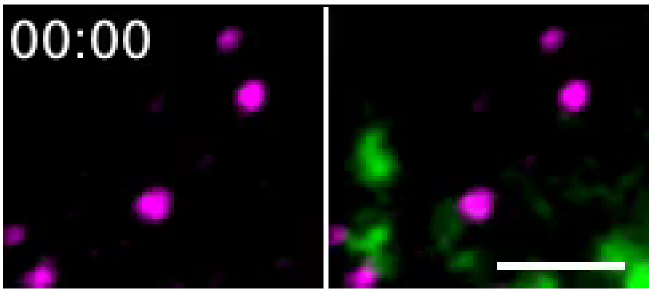

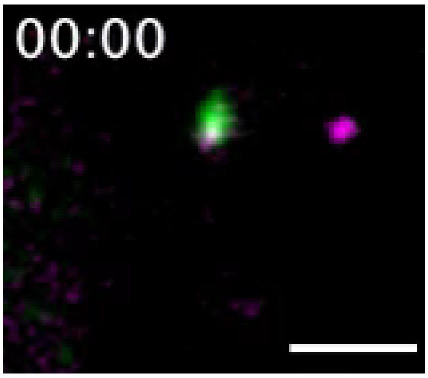

Supplementary Video S1-2 HEK293 cells expressing YFP-EEA1 (magenta) and Halo-RhoBTB3 (green) to show dynamic interactions between EEs and RhoBTB3-labeled Golgi vesicles (time interval: 8s).

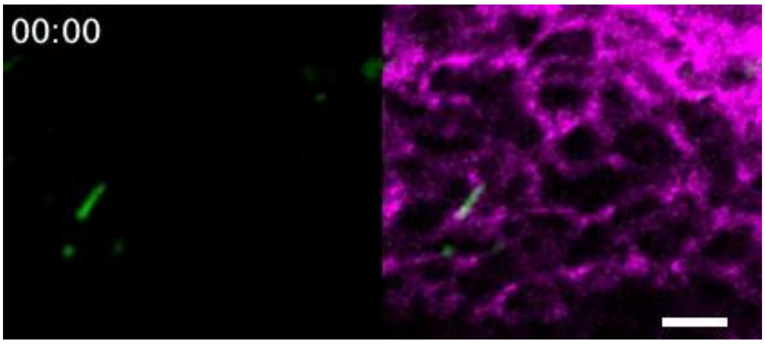

Supplementary Video S3 A HEK293 cell expressing ER-tagRFP (magenta) and Halo-RhoBTB3 (green) to show transient association between the ER and Halo-RhoBTB3-positive Golgi vesicles (time interval: 14s).

Antibodies, reagents, plasmids, siRNAs and primers used in this study

**REAGENT or RESOURCE**	Source	Catalog NO.
**Antibody**		
SHIP164	Proteintech	25121-1-AP
GFP	CST	2555
Halo	Promega	G928A
EEA1	Proteintech	28347-1-AP
VPS26B	Proteintech	15915-1-AP
RhoBTB3	Proteintech	13945-1-AP
RhoBTB3	Proteintech	67502-1-Ig
RAB14	Proteintech	15662-1-AP
TGN46	ABclonal	A19618
RAB5	Proteintech	11947-1-AP
VAPA	Proteintech	15275-1-AP
VAPB	ABclonal	A5363
GST	Proteintech	10000-0-AP
His	ABclonal	AE003
HRP-conjugated Affinipure Goat Anti-Rabbit IgG(H+L)	Proteintech	SA00001-2
HRP-conjugated Affinipure Goat Anti-Mouse IgG(H+L)	Proteintech	SA00001-1
HRP-conjugated affinipure mouse Anti-Rabbit IgG light Chain	ABcloanl	AS061

**Reagent**		
Lipofectamin2000	Invitrogen	11668019
Lipofectamin^{TM}RNAiMAX	Invitrogen	13778150
549 HaloTag Ligand	Promega	GA1110
SNAP-cell 647-SiR	NEB	S9102S
Phalloidin Alexa Fluor-594	ThermoFisher	A12381
Proteinase cocktail	Topscience	C0001
GFP-Nanoab-Agarose	LABLEAD	GNA-50-1000
Hieff clone Universal One Step Cloning Kit	YEASEN	10922ES20

**Plasmid**	**Source**
mEGFP-C1	addgene 54579
mEGFP-N1	addgene 54767
GFP-SHIP164	This study
GST-SHIP164(890-1464)	This study
GFP-SHIP164(1-266)	This study
GFP-SHIP164(266-1464)	This study
GFP-SHIP164(1190-1364)	This study
Halo-RhoBTB3	This study
Halo-VPS26B	This study
Vps26B-BFP-RFnb	This study
Arf1-Halo	This study
Arf4-Halo	This study
Arf6-Halo	This study
Halo-RHOA	This study
Halo-CDC42	This study
Halo-Rab1B	This study
Halo-RhoBTB1	This study
Halo-Rab6B	This study
Halo-RAC1	This study

Halo-VPS35	This study
Halo-VPS26A	This study
OFP-EEA1	This study
Halo-VAPA	This study
Halo-MOSPD2	This study
BFP-Rab7	This study
Halo-Rab11	This study
Halo-VAPB	This study
Halo-SMS1	This study
Halo-SMS2	This study
OFP-Rab5A	This study
Halo-Rab14	This study
Halo-Rab14 Q70L	This study
Halo-Rab14 N124I	This study
Halo-Rab10	This study
Halo-Rab4	This study
Halo-Rab21	This study
Halo-Rab17	This study
Halo-Rab22	This study
Halo-Rab23	This study
mCh-Rab45	This study
GFP-Rab7	This study
SHIP164^sfGFP (mut1)	This study
GFP-SHIP164-mut2	This study
Cor1C-GFP	This study
PTY-mch-VPS35	a gift from Yueguang Rong (HUST)
PTY-mch-VPS29	a gift from Yueguang Rong (HUST)
YFP-EEA1	a gift from Yueguang Rong (HUST)

**siRNA**	
siRNA Human scrambled siRNA:<br>5′- CGUUAAUCGCGUAUAAUACGCGUAT-3	Ribobio
siRNA Human SHIP164#1:<br>5’- AATGTCAGCTGTCTATATA -3’	Ribobio
siRNA Human SHIP164#2:<br>5’- GTCAAACAGACGTATTACT -3’	Ribobio
siRNA Human EEA1#1:<br>5’- GGGTGAAATTGCAGTATTA -3’	Ribobio
siRNA Human EEA1#2:<br>5’- CACAAGCGTTGAATAGAAA -3’	Ribobio
siRNA Human EEA1#3:<br>5’- GCATGGGTTACAACTCCAA -3’	Ribobio
siRNA Human VPS26B#1:<br>5’- CCATGAGAATGACACGATA -3’	Ribobio
siRNA Human VPS26B#2:<br>5’- GCCTTCGACTTTGAGTTTA -3’	Ribobio
siRNA Human VPS26B#3:<br>5’- GCATCAAGATCGAGTTCAT -3’	Ribobio
siRNA Human RAB5#1:<br>5’- GTCCGCTGTTGGCAAATCA -3’	Ribobio
siRNA Human RAB5#2:<br>5’- GCAAGCAAGTCCTAACATT -3’	Ribobio
siRNA Human RAB5#3:<br>5’- CAGCCATAGTTGTATATGA -3’	Ribobio

**Primers**	**Sequences (5'-3')**
GFP-SHIP164-F	tacaagtccggactcagatctGCCGGGATCATCAAGAAACA

GFP-SHIP164-R	cagaattcgaagcttgagctcCTATTCAACTGTCATCTTCTTTATATGATGA
GFP-SHIP164 (1-104AA)-F	gCTTCgAATTCTgCAgTCgACATggCCgggATCATCAAgA
GFP-SHIP164 (1-104AA)-R	CggTggATCCCgggCCCgCggTCATgATgggCCATTAgggC
GFP-SHIP164 (1-266AA)-R	CggTggATCCCgggCCCgCggCTATgATgggCCATTAgggCT
GFP-SHIP164 (700-1464AA)-F	gcttcgaattctgcagtcgacttttggatagattatgaagggatgaa
GFP-SHIP164 (700-1464AA)-R	ttatctagatccggtggatccCTAttcaactgtcatcttctttatatgatga
GFP-SHIP164 (700-1061AA)-R	ttatctagatccggtggatccctaGTTTTCAGAGAGTGAAGCTGCTTCT
GFP-SHIP164 (1062-1464AA)-F	gcttcgaattctgcagtcgacCTGGATATCAGTAAAGAAGAGACCCC
GFP-SHIP164 (1365-1464AA)-F	gcttcgaattctgcagtcgacCTTAACACTGGAAATGATTTGAAAGAA
GST-SHIP164 (890-1464AA)-F	GATCTGGTTCCGCGTGGATCCagcccagtggtacctgattatttg
GST-SHIP164 (890-1464AA)-R	TCAGTCAGTCACGATGAATTCctattcaactgtcatcttctttatatgatga
Halo-SHIP164 (1-104AA)-F	ctggagatttccctcgagctcatggccgggatcatcaaga
Halo-SHIP164 (1-104AA)-R	gtaccgtcgactgcagaattcTCAtgatgggccattagggc
GFP-SHIP164 (delete 1-104AA)-F	gactcagatctatgccaattgcaactgcttcagga
GFP-SHIP164 (delete 1-104AA)-R	tggcatagatctgagtccggacttgtacagct
GFP-SHIP164 (delete 1-266AA)-F	gCTTCgAATTCTgCAgTCgACATgTCAACAgAACAAAggAAgAgTATg
GFP-SHIP164 (delete 1-266AA)-R	CggTggATCCCgggCCCgCggCTATTCAACTgTCATCTTCTTTATATgATgA
GFP-SHIP164 (1-104AA Mut1-2)-F	agGCgtgcttcaagTCttgtttcttgatgatcccggc
GFP-SHIP164 (1-104AA Mut1-2)-R	GACAAGgacaatgcaAGTACCCTTAAAGGAGAAGGTGAAGC
GFP-SHIP164 (1-104AA Mut1-4)-F	GACAAGgacaatgcaAGTACCCTTAAAGGAGAAGGTGAAGC
GFP-SHIP164 (1-104AA Mut1-4)-R	cttGCattGTCcttgtcaggagataaatttttggtaaa
GFP-SHIP164 (1-104AA Mut1-6)-F	GCgaagaatttggagGCggatgaagaagtactccagaatatgttgg
GFP-SHIP164 (1-104AA Mut1-6)-R	cGCctccaaattcttcGCttcaccttctcctttaagggtactt
GFP-SHIP164 (1-104AA Mut7-8)-F	ttgtaataaagcgtccgatAGGATCCCATGGACAAAACTGA
GFP-SHIP164 (1-104AA Mut7-8)-R	CggacgctttattacaaaaaactttgttgTCagcaagccatgttggcaaat
Halo-RhoBTB3-F	CTggAgATTTCCCTCgAgCTCATgTCCATCCACATCgTggC
Halo-RhoBTB3-R	ttatctagatccggtggatccTTACATTACTAAGCAACGACATTTCCG
SNAP-RhoBTB3-F	cctgggctgggtctcgagctcatgtccatccacatcgtggc
GFP-RhoBTB3-F	gcttcgaattctgcagtcgacatgtccatccacatcgtggc
Halo-RhoBTB3 (N138D)-F	CCAgACAAgATgAAgAgTTACCTTgTACATgCCCA
Halo-RhoBTB3 (N138D)-R	CTCTTCATCTTgTCTggTACCAACAgCAgCAA
Halo-RhoBTB3 (A498T)-F	CCTgATCTgTACAgAgATgTACCAAgTgTCCAgACTgC
Halo-RhoBTB3 (A498T)-R	TCTCTgTACAgATCAggAgACACATggCCTgg
Halo-RhoBTB3 (D532E)-F	CCTTgAAATAgTTgACCTgCTTAAAAAggCCA
Halo-RhoBTB3 (D532E)-R	ggTCAACTATTTCAAggTTCATggATgCCAgTTCC
Halo-RhoBTB3 (I533K)-F	CCTTgATAAAgTTgACCTgCTTAAAAAggCCA
Halo-RhoBTB3 (I533K)-R	ggTCAACTTTATCAAggTTCATggATgCCAgT
Halo-VPS26B-F	ctgcagtcgacggtaccgcggATGAGCTTCTTCGGCTTCGG
Halo-VPS26B-R	ttatctagatccggtggatccCTACTGCCTGCAGTTGTTGTCAG
OFP-VPS26B Δ (1-160AA)-F	ctgcagtcgacggtaccgcggTCCATCAAGATGGAGGTTGGG
OFP-VPS26B Δ (161-336AA)-R	ttatctagatccggtggatccttaAGAGTTCAGCTCTGGGTATGTGC
Arf1-Halo-F	tgaaccgtcagatccgctagcATGGGGAACATCTTCGCCA
Arf-Halo-R	cggtggatcccgggcccgcggCTTCTGGTTCCGGAGCTGATT

Arf4-Halo-F	tgaaccgtcagatccgctagcATGGGCCTCACTATCTCCTCCC
Arf4-Halo-R	cgactgcagaattcgaagcttACGTTTTGAAAGCTCATTTGACAG
Arf6-Halo-F	tgaaccgtcagatccgctagcGCCACCATGGGGAAGGTGCTATCCAAA
Arf6-Halo-R	cgactgcagaattcgaagcttAGATTTGTAGTTAGAGGTTAACCATGTGA
Halo-RHOA-F	ctggagatttccctcgagctcGCTGCCATCCGGAAGAAAC
Halo-RHOA-R	ttatctagatccggtggatccTCACAAGACAAGGCACCCAGA
Halo-CDC42-F	ctggagatttccctcgagctcACGGCGGCCCAGGCCGCG
Halo-CDC42-R	ttatctagatccggtggatccTCAGGTCACCACGCAAAAGC
Halo-Rab1B-F	ctggagatttccctcgagctcATGAACCCCGAATATGACTACCTG
Halo-Rab1B-R	ttatctagatccggtggatccCTAGCAACAGCCACCGCCA
Halo-RhoBTB1-F	ctggagatttccctcgagctcGACGCTGACATGGACTACGAAA
Halo-RhoBTB1-R	ttatctagatccggtggatccTCAGGCCACTGCTGGAGATG
Halo-Rab6B-F	atttccctcgagctcaagcttATGTCCGCAGGGGGAGATT
Halo-Rb6B-R	ttatctagatccggtggatccTTAGCAGGAGCAGCCGCC
Halo-RAC1-F	ctggagatttccctcgagctcATGCAGGCCATCAAGTGTGTG
Halo-RAC1-R	ttatctagatccggtggatccTTACAACAGCAGGCATTTTCTCTT
Halo-VPS35-F	ctggagatttccctcgagctcATGCCTACAACACAGCAGTCCC
Halo-VPS35-R	gtaccgtcgactgcagaattcTTAAAGGATGAGACCTTCATAAATTGG
Halo-VPS26A-F	ctgcagtcgacggtaccgcggATGAGTTTTCTTGGAGGCTTTTTT
Halo-VPS26A-R	ttatctagatccggtggatccctaCATTTCAGGCTGTTCGGCA
OFP-EEA1-F	ctgcagtcgacggtaccgcggTGGCAATCTAGTCAACGGAGAGT
OFP-EEA1-R	ttatctagatccggtggatccTTATCCTTGCAAGTCATTGAAACAT
Halo-VAPA-F	ctggagatttccctcgagctcATGGCGTCCGCCTCAGGG
Halo-VAPA-R	ttatctagatccggtggatccCTACAAGATGAATTTCCCTAGAAAGAAT
Halo-MOSPD2-F	ctggagatttccctcgagctcATGGCAGAGAATCACGCCC
Halo-MOSPD2-R	ttatctagatccggtggatccTTAACTGTACAATAAATAGAAGAAAGAGGTG
BFP-Rab7A-F	agatccgctagcgctaccggtATGAGCGAGCTGATTAAGGAGAA
BFP-Rab7A-R	cctagaggtcattcgagatctATTAAGCTTGTGCCCCAGTTTG
Halo-Rab11-F	ctggagatttccctcgagctcATGGGCACCCGCGACGAC
Halo-Rab11-R	ttatctagatccggtggatccTTAGATGTTCTGACAGCACTGCACC
Halo-SMS1-F	ctgcagtcgacggtaccgcggATGAAGGAAGTGGTTTATTGGTCAC
Halo-SMS1-R	ttatctagatccggtggatccTTATGTGTCATTCACCAGCCGG
Halo-SMS2-F	ctgcagtcgacggtaccgcggATGGATATCATAGAGACAGCAAAACTTG
Halo-SMS2-R	ttatctagatccggtggatcctcaGGTCGATTTCTCATTGTCTTCACC
CERS2-OFP-F	ctaccggactcagatctcgagGCCACCATGCTCCAGACCTTGTATGATTACTT
CERS2-OFP-R	cgttctcagtgctatcAAGCTTGTCATTCTTACGATGGTTGTTATTGAG
OFP-Rab5A-F	ctgcagtcgacggtaccgcggATGGCTAGTCGAGGCGCAA
OFP-Rab5A-R	ttatctagatccggtggatccTTAGTTACTACAACACTGATTCCTGGTTG
Halo-Rab14-F	ctgcagtcgacggtaccgcggATGGCAACTGCACCATACAACTA
Halo-Rab14-R	ttatctagatccggtggatccCTAGCAGCCACAGCCTTCTCTC
Halo-Rab14-Q70L-F	GGCAGGAttaGAGCGATTTAGGGCTGTTACACG
Halo-Rab14-Q70L-R	ATCGCTCtaaTCCTGCCGTATCCCAAATCTGC
Halo-Rab14-N124I-F	CATAGGAatcAAAGCAGATTTGGAGGCACAGA
Halo-Rab14-N124I-R	CTGCTTTgatTCCTATGAGAATTATTACAGTATTTGGATT
Halo-Rab4-F	ctggagatttccctcgagctcATGTCGCAGACGGCCATG
Halo-Rab4-R	ttatctagatccggtggatccCTAACAACCACACTCCTGAGCG

Halo-Rab21-F	ctggagatttccctcgagctcATGGCTGCGGCCGGCGGC
Halo-Rab21-R	ttatctagatccggtggatccTTATCCAGAAGAACAGCACCCTC
Halo-Rab17-F	ctggagatttccctcgagctcATGGCACAGGCACACAGGAC
Halo-Rab17-R	ttatctagatccggtggatccCTAGTGGGCGCAGCATTTG
Halo-Rab22-F	ctggagatttccctcgagctcATGGCGCTGAGGGAGCTC
Halo-Rab22-R	ttatctagatccggtggatccTCAGCAGCAGCTCCGCTT
Halo-Rab23-F	ctggagatttccctcgagctcATGTTGGAGGAAGATATGGAAGTCG
Halo-Rab23-R	ttatctagatccggtggatccTTAGGGTATGCTACAGCTGCTAAAA
GFP-Rab7-F	agtccggactcagatctcgaatgacctctaggaagaaagtgttgc
GFP-Rab7-R	gtaccgtcgactgcagaattctcagcaactgcagctttctgc
mCh-Rab45-F	ggcaatgcctgctgggagctcATGGAGGCGGATGGGGAC
mCh-Rab45-R	ttatctagatccggtggatccTTAGCCATTGCAACAATTCTTCA
SHIP164^sfGFP-1-F	ctgcagtcgacggtaccgcggATGGCCGGGATCATCAAGAAACA
SHIP164^sfGFP-1-R	tctcctttgctcatgctgcttccgctgcttccATAGACAGCTGACATTTCTTGTGGA
SHIP164^sfGFP-2-F	gctgtctatGGAAGCAGCGGAAGCAGCatgAGCAAAGGAGAAGAACTTTTCACTG
SHIP164^sfGFP-2-R	TTTGTAGAGCTCATCCATGCCA
SHIP164^sfGFP-3-F	gcatggatgagctctacaaaGGAAGCAGCGGAAGCAGCATAGAATTCACAGAATATTACTATCCAGATGG
SHIP164^sfGFP-3-R	ttatgatctagagtcgcggccgcctaTTCAACTGTCATCTTCTTTATATGATGAA
Cor1C-GFP-F	tgaaccgtcagatccgctagcGCCACCATGAGGCGAGTGGTACGACAG
Cor1C-GFP-R	cgactgcagaattcgaagcttGGCTGCTATCTTTGCCATCTG
SHIP164 KO sgRNA-1	GTACACAGCCCAAACATCCG
SHIP164 KO sgRNA-2	AGGTCAGAATGTCGACAGTT
verify SHIP164 KO-F	ATCTTCAAGATGTATGGATTTGCCA
verify SHIP164 KO-R	ACTGACATGTTTATGAACATGAACTAG

Raw WB images in Fig. 1

Panel c

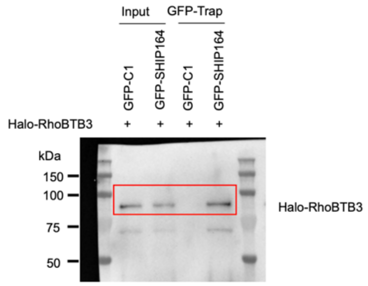

Panel c

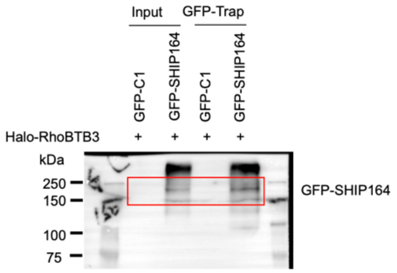

Panel c

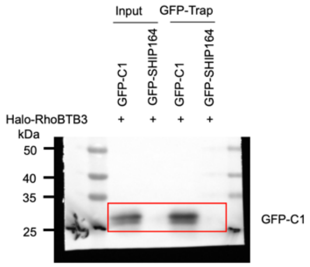

Panel d

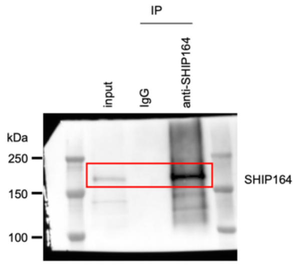

Panel d

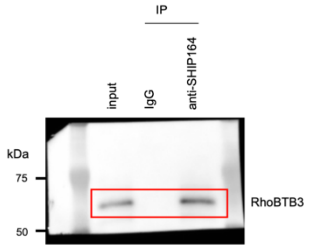

Panel d

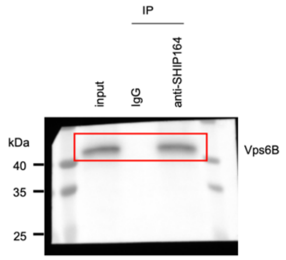

Panel e

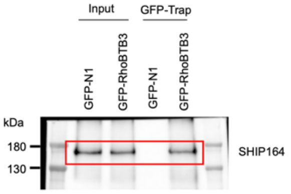

Panel e

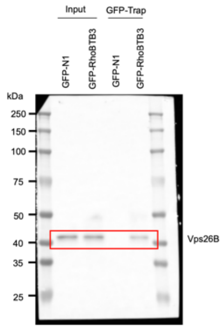

Panel e

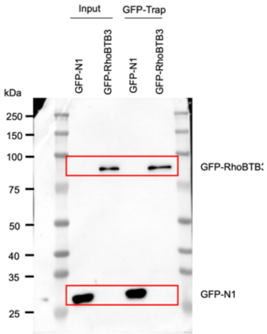

Panel q

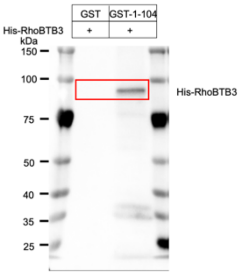

Panel q

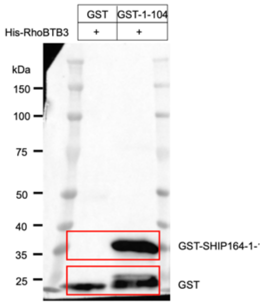

Raw WB images in Fig. 2

Panel c

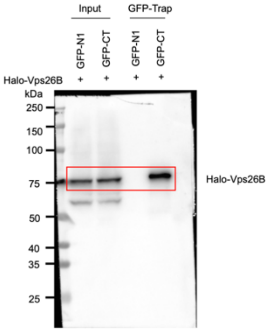

Panel c

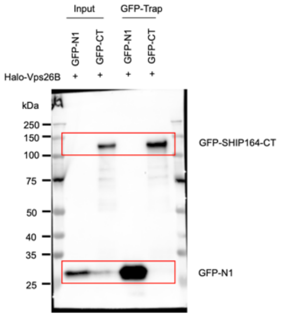

Panel d

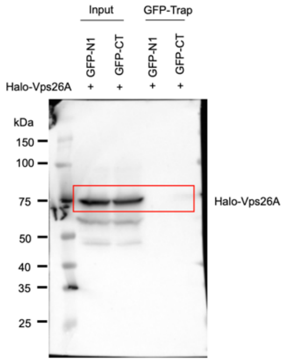

Panel d

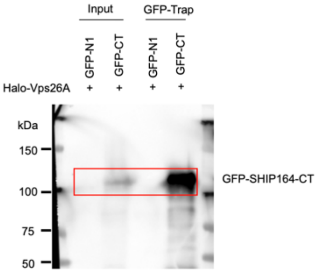

Panel d

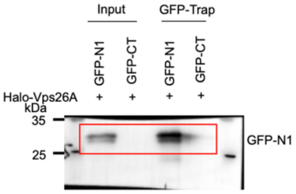

Panel e

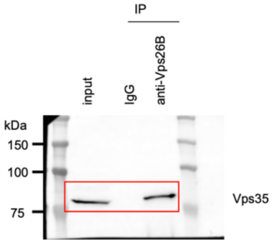

Panel e

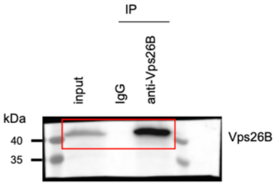

Panel e

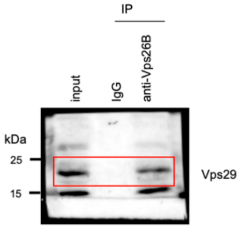

Panel h

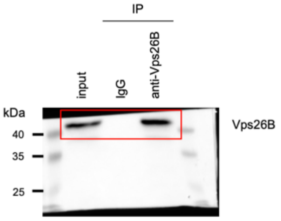

Panel h

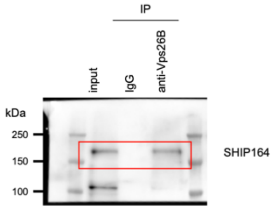

Panel i

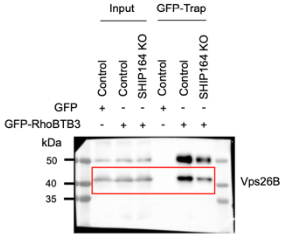

Panel i

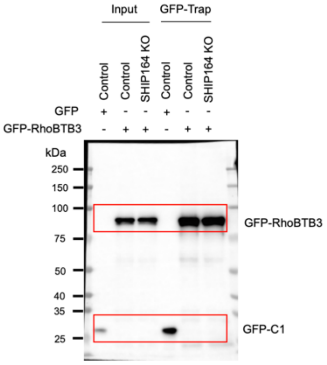

Panel s

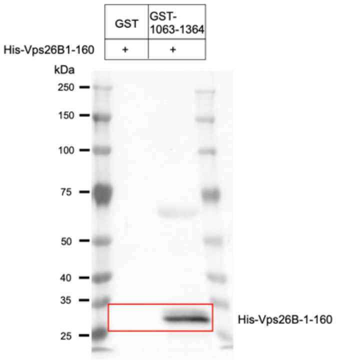

Panel s

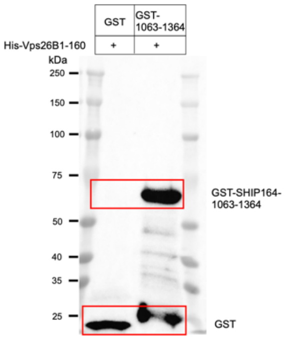

Raw WB images in Fig. 3

Panel e

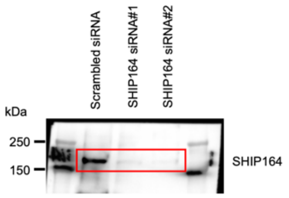

Panel e

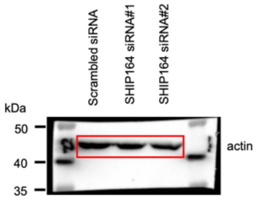

Raw WB images in Fig. 4

Panel l

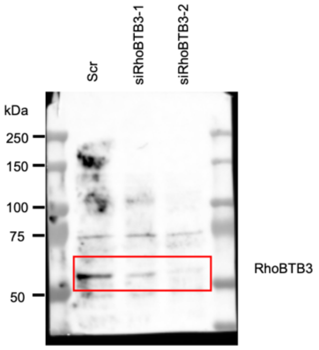

Panel l

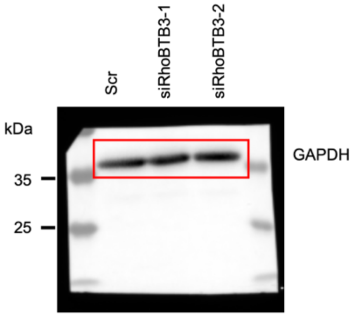

Panel m

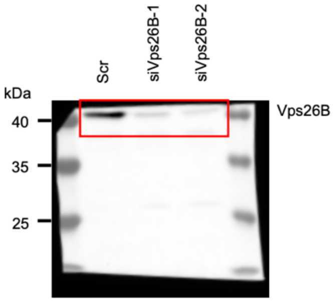

Panel m

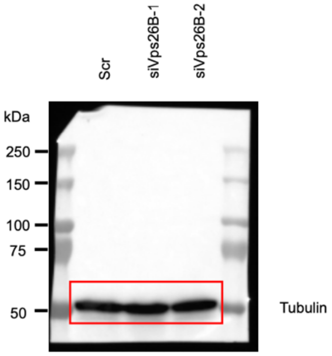

Raw WB images in Fig. 5

Panel h

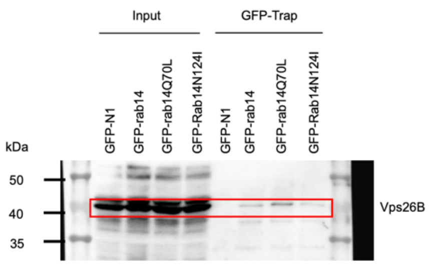

Panel h

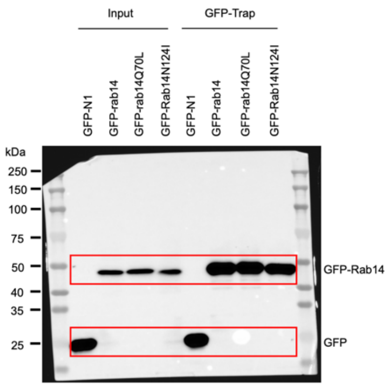

Panel i

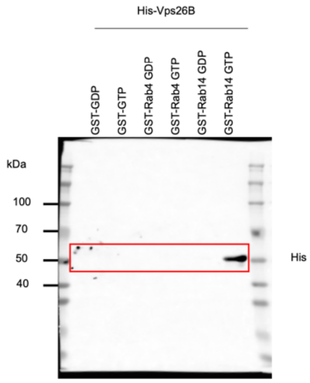

Panel i

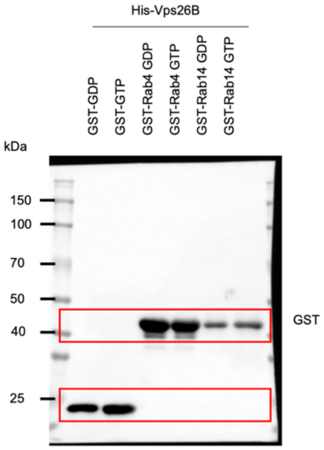

Panel j

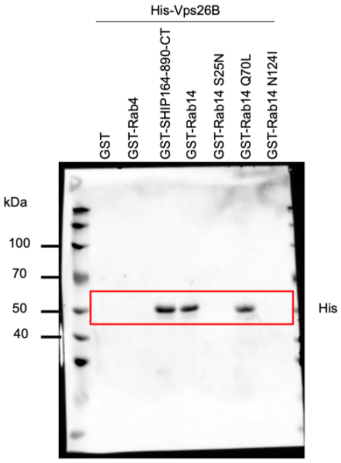

Panel j

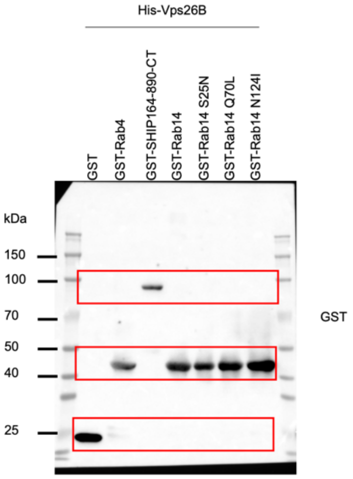

Raw WB images in Fig. 6

Panel b

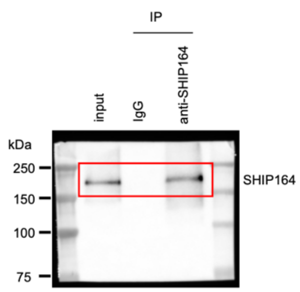

Panel b

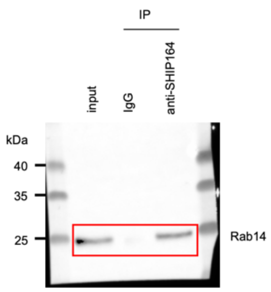

Panel f

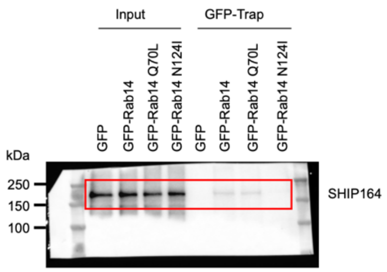

Panel f

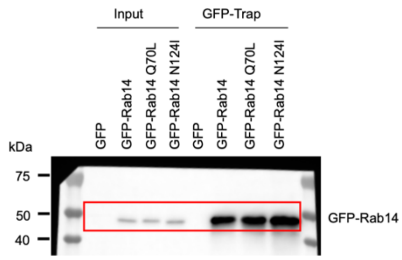

Panel f

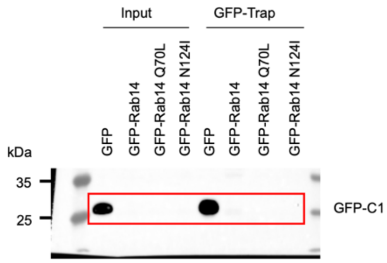

Panel g

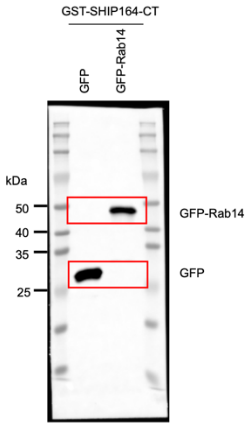

Panel g

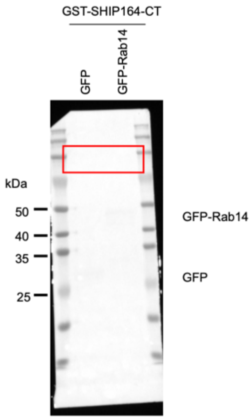

Raw WB images in Fig. 7

Panel i

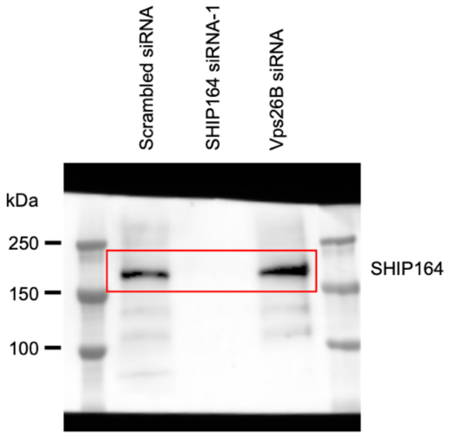

Panel i

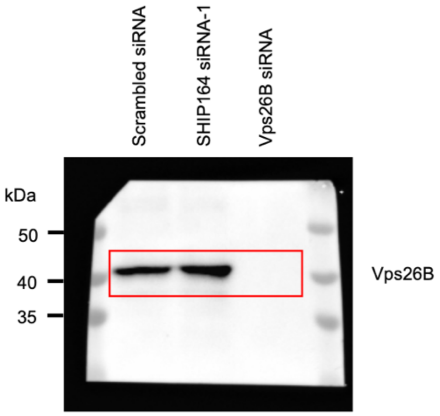

.

Panel i

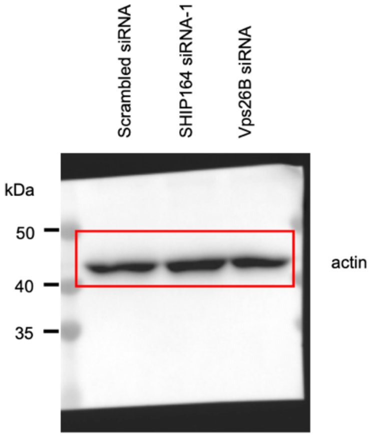

Raw WB images in Supplementary Fig. 1

Panel c

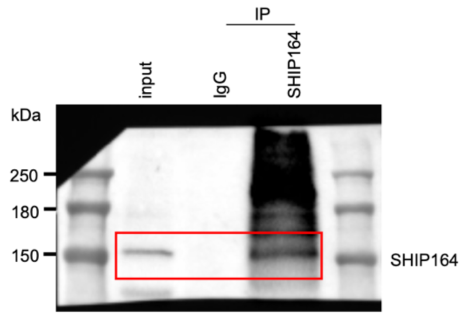

Panel c

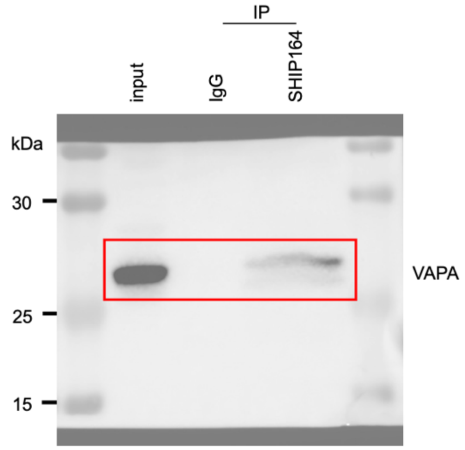

Panel c

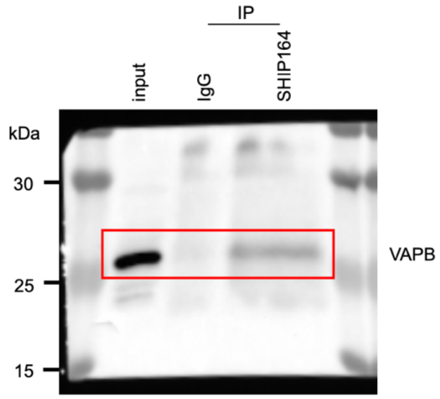

Raw WB images in Supplementary Fig. 4

Panel b

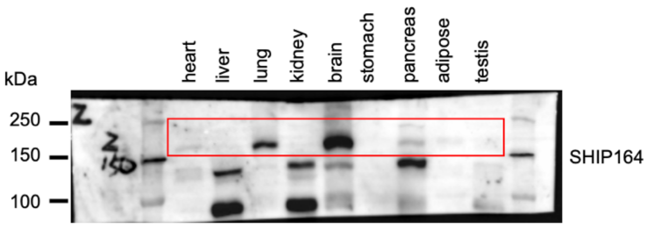

Panel b

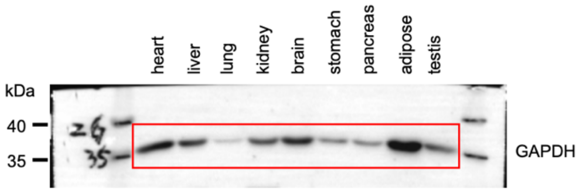

Raw WB images in Supplementary Fig. 5

Panel d

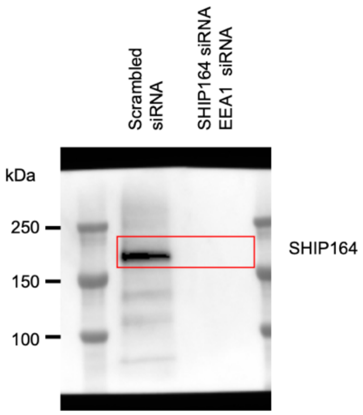

Panel d

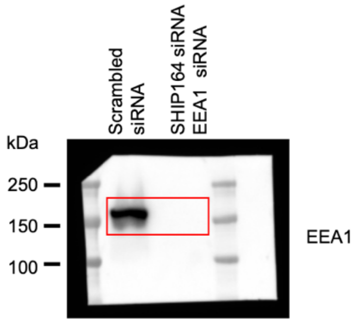

Panel d

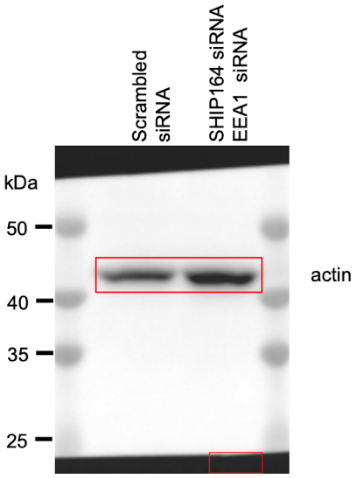

Raw WB images in Supplementary Fig. 7

Panel d

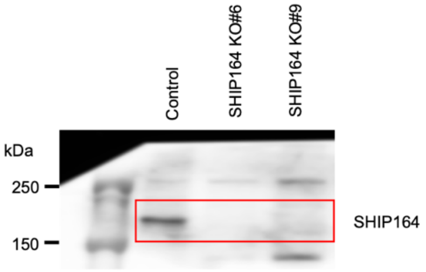

Panel d

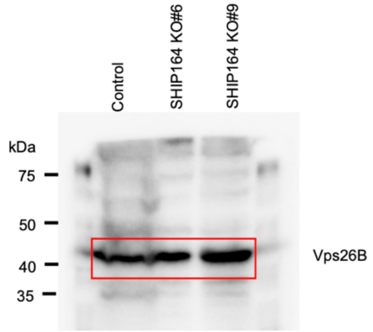

Panel d

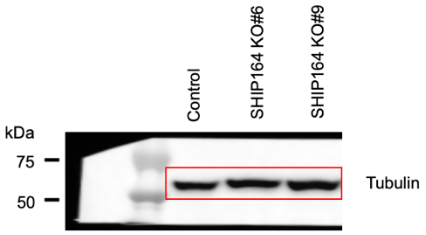

Raw WB images in Supplementary Fig.8

Panel j

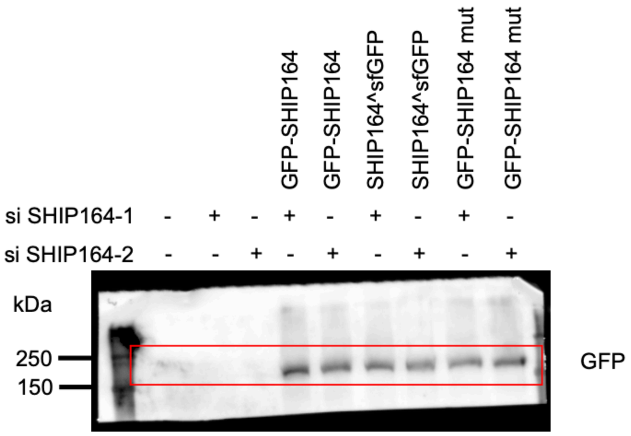

Panel j

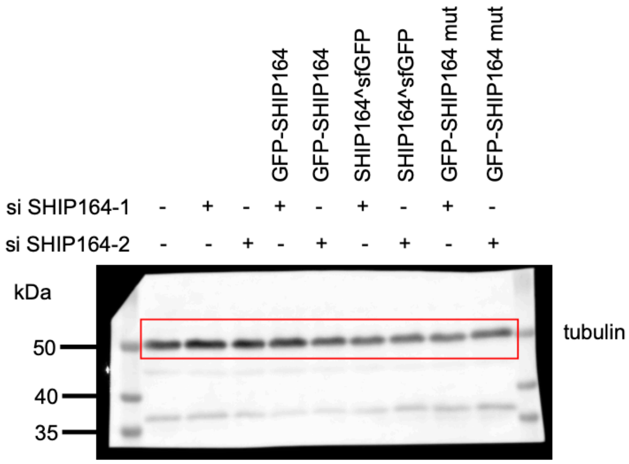

Supplement: Supplementary file 1 — Supplementary Information [file 41421_2024_651_MOESM1_ESM.pdf]
